# Tuberculosis screening among HIV-positive inpatients: a systematic review and individual participant data meta-analysis

**DOI:** 10.1016/S2352-3018(22)00002-9

**Published:** 2022-03-23

**Authors:** Ashar Dhana, Yohhei Hamada, Andre P Kengne, Andrew D Kerkhoff, Molebogeng X Rangaka, Tamara Kredo, Annabel Baddeley, Cecily Miller, Ankur Gupta-Wright, Katherine Fielding, Robin Wood, Helena Huerga, Sekai Chenai Mathabire Rücker, Christine Heidebrecht, Douglas Wilson, Stephanie Bjerrum, Isik S Johansen, Swe Swe Thit, Mar Mar Kyi, Josh Hanson, David A Barr, Graeme Meintjes, Gary Maartens

**Affiliations:** a Department of Medicine, University of Cape Town, Cape Town, South Africa; b Wellcome Centre for Infectious Diseases Research in Africa, Institute of Infectious Disease and Molecular Medicine, Faculty of Health Sciences, University of Cape Town, Cape Town, South Africa; c Centre for International Cooperation and Global TB Information, The Research Institute of Tuberculosis, Japan Anti-Tuberculosis Association, Tokyo, Japan; d Institute for Global Health, University College London, London, UK; e Non-communicable Diseases Research Unit, South African Medical Research Council, Cape Town, South Africa; f Cochrane South Africa, South African Medical Research Council, Cape Town, South Africa; g Division of HIV, Infectious Diseases and Global Medicine, Zuckerberg San Francisco General Hospital and Trauma Center, University of California San Francisco, San Francisco, CA, USA; h Global TB Programme, WHO, Geneva, Switzerland; i Clinical Research Department, London School of Hygiene and Tropical Medicine, London, UK; j TB Centre, London School of Hygiene and Tropical Medicine, London, UK; k Field Epidemiology Department, Epicentre, Paris, France; l Institute for Better Health, Trillium Health Partners, Mississauga, ON, Canada; m Department of Internal Medicine, Edendale Hospital, University of KwaZulu-Natal, Pietermaritzburg, South Africa; n Research Unit for Infectious Diseases, Odense University Hospital, University of Southern Denmark, Odense, Denmark; o Department of Medicine, University of Medicine, Yangon, Myanmar; p The Kirby Institute, University of New South Wales, Sydney, NSW, Australia; q Institute of Infection and Global Health, University of Liverpool, Liverpool, UK

## Abstract

**Background:**

Since 2011, WHO has recommended that HIV-positive inpatients be routinely screened for tuberculosis with the WHO four-symptom screen (W4SS) and, if screened positive, receive a molecular WHO-recommended rapid diagnostic test (eg, Xpert MTB/RIF [Xpert] assay). To inform updated WHO tuberculosis screening guidelines, we conducted a systematic review and individual participant data meta-analysis to assess the performance of W4SS and alternative screening tests to guide Xpert testing and compare the diagnostic accuracy of the WHO Xpert algorithm (ie, W4SS followed by Xpert) with Xpert for all HIV-positive inpatients.

**Methods:**

We searched MEDLINE, Embase, and Cochrane Library from Jan 1, 2011, to March 1, 2020, for studies of adult and adolescent HIV-positive inpatients enrolled regardless of tuberculosis signs and symptoms. The separate reference standards were culture and Xpert. Xpert was selected since it is most likely to be the confirmatory test used in practice. We assessed the proportion of inpatients eligible for Xpert testing using the WHO algorithm; assessed the accuracy of W4SS and alternative screening tests or strategies to guide diagnostic testing; and compared the accuracy of the WHO Xpert algorithm (W4SS followed by Xpert) with Xpert for all. We obtained pooled proportion estimates with a random-effects model, assessed diagnostic accuracy by fitting random-effects bivariate models, and assessed diagnostic yield descriptively. This systematic review has been registered on PROSPERO (CRD42020155895).

**Findings:**

Of 6162 potentially eligible publications, six were eligible and we obtained data for all of the six publications (n=3660 participants). The pooled proportion of inpatients eligible for an Xpert was 90% (95% CI 89–91; n=3658). Among screening tests to guide diagnostic testing, W4SS and C-reactive protein (≥5 mg/L) had highest sensitivities (≥96%) but low specificities (≤12%); cough (≥2 weeks), haemoglobin concentration (<8 g/dL), body-mass index (<18·5 kg/m^2^), and lymphadenopathy had higher specificities (61–90%) but suboptimal sensitivities (12–57%). The WHO Xpert algorithm (W4SS followed by Xpert) had a sensitivity of 76% (95% CI 67–84) and specificity of 93% (88–96; n=637). Xpert for all had similar accuracy to the WHO Xpert algorithm: sensitivity was 78% (95% CI 69–85) and specificity was 93% (87–96; n=639). In two cohorts that had sputum and non-sputum samples collected for culture or Xpert, diagnostic yield of sputum Xpert was 41–70% and 61–64% for urine Xpert.

**Interpretation:**

The W4SS and other potential screening tests to guide Xpert testing have suboptimal accuracy in HIV-positive inpatients. On the basis of these findings, WHO now strongly recommends molecular rapid diagnostic testing in all medical HIV-positive inpatients in settings where tuberculosis prevalence is higher than 10%.

**Funding:**

World Health Organization.

## Introduction

Tuberculosis is the leading cause of hospital admission and in-hospital deaths in people living with HIV.^1^ In a meta-analysis of autopsy studies among people living with HIV, almost 50% of tuberculosis-related deaths were undiagnosed at autopsy.^2^ The diagnosis of tuberculosis among HIV-positive inpatients is challenging. HIV-positive inpatients are typically severely immune suppressed with disseminated or extrapulmonary tuberculosis, might have a non-specific clinical presentation, and often produce paucibacillary specimens.^3^ Furthermore, a large proportion (31–63%) of inpatients are unable to produce sputum for diagnostic testing.^4^, ^5^, ^6^

Since 2011, WHO has recommended that people living with HIV (including HIV-positive inpatients) be routinely screened for tuberculosis with the WHO four-symptom screen (W4SS; comprising current cough, fever, night sweats, or weight loss);^7^ if the W4SS is positive, an inpatient should then receive a molecular WHO-recommended rapid diagnostic test (eg, Xpert MTB/RIF [Xpert] or Xpert MTB/RIF Ultra [Xpert Ultra]).^8^

**Research in context**

**Evidence before this study**

Tuberculosis is the leading cause of hospital admission and in-hospital death among people living with HIV and often goes undiagnosed. Since 2011, WHO has recommended that people living with HIV (including HIV-positive inpatients) be routinely screened for tuberculosis with the WHO four-symptom screen (W4SS; comprising any one symptom of current cough, fever, night sweats, or weight loss); if the W4SS is positive, a molecular WHO-recommended rapid diagnostic test (eg, Xpert MTB/RIF [Xpert] or Xpert MTB/RIF Ultra [Xpert Ultra]) should be done. However, inpatients with other HIV-related opportunistic diseases often have a positive W4SS. Furthermore, the diagnostic accuracy of alternative screening tests and strategies in HIV-positive inpatients is unclear. To inform updated WHO tuberculosis screening guidelines, we did an individual participant data meta-analysis among HIV-positive inpatients who were enrolled regardless of tuberculosis signs and symptoms. We searched MEDLINE, Embase, and Cochrane Library from Jan 1, 2011, to March 1, 2020, with search terms related to “human immunodeficiency virus”, “tuberculosis”, “screening”, “algorithm”, “sensitivity”, and “specificity”. We calculated the proportion of inpatients eligible for Xpert testing using the WHO algorithm (W4SS followed by Xpert); assessed the accuracy of W4SS and alternative screening tests and strategies to guide diagnostic testing; and compared the accuracy of the WHO Xpert algorithm (W4SS followed by Xpert) with Xpert for all.

**Added value of this study**

We used individual participant data from six studies of HIV-positive medical inpatients admitted to hospital irrespective of tuberculosis signs and symptoms. We found that 90% of all HIV-positive inpatients were eligible for Xpert testing using the WHO algorithm. Among screening tests to guide additional diagnostic testing, the W4SS and C-reactive protein concentration (≥5 mg/L) had the highest sensitivities, but low specificities. Cough (≥2 weeks), haemoglobin concentration (<8 g/dL), body-mass index (<18·5 kg/m2), and lymphadenopathy had low sensitivities and moderate-to-high specificities. The WHO Xpert algorithm (W4SS followed by Xpert) had a sensitivity of 76% and Xpert for all had a similar sensitivity (78%). Sputum Xpert alone had low yield, mostly because a high proportion of inpatients were unable to produce sputum.

**Implications of all the available evidence**

On the basis of these findings, WHO has made a strong recommendation to do molecular rapid diagnostic testing (eg, with Xpert) for tuberculosis in all HIV-positive medical inpatients in high-burden settings (>10% tuberculosis prevalence). Molecular rapid diagnostic testing in all HIV-positive medical inpatients would reduce the current diagnostic gap; however, a negative result does not rule out tuberculosis.

Rapid tuberculosis diagnostic testing with Xpert in all HIV-positive inpatients in high-burden settings might be more appropriate than pre-screening with the W4SS to assess eligibility for Xpert testing. The W4SS was developed following an individual participant data meta-analysis in ambulatory patients with HIV.^7^ However, W4SS might have low specificity in inpatients since HIV-related opportunistic diseases often present with one or more of the W4SS symptoms.^9^ Furthermore, the diagnostic accuracies of alternative screening tests or strategies are not well known.

We conducted a systematic review and individual participant data meta-analysis of HIV-positive inpatients admitted to hospital irrespective of signs and symptoms of tuberculosis to inform updated WHO tuberculosis screening guidelines.^10^ First, we calculated the proportion of inpatients eligible for rapid tuberculosis diagnostic testing with Xpert using the WHO algorithm (W4SS followed by Xpert). Second, we assessed the diagnostic accuracy of the W4SS and other tuberculosis screening tests or strategies to guide diagnostic testing. Third, we compared the diagnostic accuracy of the WHO Xpert algorithm (W4SS followed by Xpert) with Xpert for all inpatients. Fourth, we calculated the diagnostic yield of Xpert (ie, proportion of total tuberculosis cases with a positive Xpert test).

## Methods

### Search strategy and selection criteria

We used similar methods to our recent individual participant data meta-analysis on tuberculosis screening among people living with HIV who were in ambulatory care.^11^ Two authors (AD and YH) independently selected studies, extracted data, and assessed study quality. Disagreements were resolved by discussion.

We updated the search of the systematic review by Hamada and colleagues,^12^ who searched PubMed (MEDLINE), Embase, Cochrane Library, and conference abstracts from Jan 1, 2011 (the year WHO first recommended the W4SS be used), to March 12, 2018 (appendix p 2). We re-reviewed all potential full-texts from Hamada and colleagues^12^ to identify eligible studies. We also applied the same search strategy from Hamada and colleagues^12^ for articles published between March 12, 2018, and March 1, 2020. We also reviewed reference lists of reviews and included articles, and we contacted experts for unpublished studies.

We reviewed titles and abstracts from the search and reviewed full-texts of potentially eligible articles. We included cross-sectional studies, observational studies, and randomised trials that collected at least one sputum sample for tuberculosis culture or Xpert among adult and adolescent (aged 10 years or older) inpatients who were HIV-positive and who were enrolled regardless of tuberculosis signs and symptoms (but with data on W4SS). The target condition was active tuberculosis.

The two separate reference standards were bacteriological confirmation of *Mycobacterium tuberculosis* with culture of sputum or other samples, or Xpert of sputum or other samples. Xpert was selected post hoc despite its suboptimal sensitivity because it is recommended by WHO and is the most used confirmatory test in practice (as opposed to culture). WHO recommends assessing screening or triage tests against currently recommended confirmatory tests.^13^ Only studies that collected culture contributed to the WHO guidelines^10^ with two additional studies added to this meta-analysis after guideline development.^4^, ^14^

We included primary datasets that allowed us to compare the W4SS with alternative screening tests and strategies, and the WHO Xpert algorithm (W4SS followed by Xpert) with rapid tuberculosis diagnostic testing in all HIV-positive inpatients using Xpert. In this Article, a screening test was defined as a test done to assess whether an inpatient requires additional testing for bacteriological confirmation of tuberculosis (eg, with a rapid molecular diagnostic test or culture) and a diagnostic test was defined as a test that would provide bacteriological confirmation. The systematic screening tests we examined were the W4SS, C-reactive protein concentration (CRP; 10 mg/L [considered the upper limit of normal],^15^ 5 mg/L, and 8 mg/L thresholds), chest x-ray, cough lasting 2 weeks or more, haemoglobin concentration (<10 g/dL and <8 g/dL), body-mass index (<18.5 kg/m^2^), and lymphadenopathy. We also examined several parallel strategies (two screening tests offered at the same time) to improve sensitivity and sequential strategies (second screening test offered only if the first screening test is positive) to improve specificity. Finally, the systematic rapid tuberculosis diagnostic tests that we examined were Xpert and Xpert Ultra (although no included study assessed Xpert Ultra).

We excluded studies that had a case-control design, that only recruited HIV-positive inpatients with presumptive tuberculosis, and that recruited participants who were already on tuberculosis treatment or currently diagnosed with active tuberculosis.

We have reported our findings according to PRISMA-IPD and PRISMA-DTA statements.^16^, ^17^ The protocol for this study was approved by the University of Cape Town human research ethics committee. For each included study, participants gave written informed consent and investigators obtained ethics committee approval.

### Data extraction, study quality, and individual participant data synthesis

Using a standardised data extraction form, we extracted study-level information on first author, publication year, study period, country, setting, exclusion criteria, study design, type of participants, and method of tuberculosis diagnosis. To assess quality of studies included in proportion meta-analyses, we modified a tool designed to assess study quality in systematic reviews addressing prevalence measures.^18^ To assess quality of diagnostic test accuracy studies, we used the Quality Assessment of Diagnostic Accuracy Studies-2 tool to assess patient selection, index test, reference standard, and flow and timing.^19^

We emailed authors of eligible datasets with an invitation to contribute individual participant data. After consultation with WHO and our study group, we prespecified variables to be collected (appendix p 3). We standardised individual participant data and then synthesised a single dataset. We excluded study participants younger than 10 years and considered contaminated cultures as negative. We ensured individual participant data integrity for each dataset by checking information against study publications and checking for missing, duplicate, invalid, and implausible items.^20^, ^21^ We contacted the corresponding authors of each study to resolve discrepancies.

### Data analyses

We analysed individual participant data in two-stages. Individual participant data were first analysed separately in each study and reduced to aggregate data, which we then pooled using meta-analytical techniques.

First, we estimated tuberculosis prevalence, proportion of inpatients eligible for Xpert testing according to the WHO algorithm (ie, proportion of inpatients with a positive W4SS), and measures of diagnostic performance (eg, sensitivity and specificity) for individual studies. Second, we pooled tuberculosis prevalence and proportion of inpatients eligible for Xpert testing using a generalised linear mixed model with logit transformation.^22^ We assessed heterogeneity with Cochran's Q test and *I*^2^ statistic.^23^ We pooled measures of diagnostic performance (sensitivity and specificity) in a bivariate generalised linear mixed model.^24^ For these analyses, we excluded HIV-positive inpatients with no data on the reference standard or index test. When there were fewer than four studies or non-convergence, we assumed no correlation between measures of sensitivity and specificity to simplify the model.^25^ When all studies had 100% sensitivity or specificity, we computed binomial 95% CIs. We showed the absolute pooled sensitivity and specificity using summary receiver-operating characteristic curves.^26^ To compare test accuracy, we did indirect comparisons (based on all studies that evaluated at least one of the tests of interest) and direct comparisons (based on studies that evaluated both tests of interest). For direct comparisons, we did a bivariate meta-regression with test-type as a covariate. Due to the variation of tuberculosis prevalence across studies, we applied pooled accuracy estimates to a hypothetical cohort of 1000 individuals for each screening test or strategy using Bayes' theorem. We also calculated the diagnostic yield of Xpert using a post-hoc analysis; diagnostic yield of Xpert was defined as the proportion of total microbiologically confirmed tuberculosis cases (using culture or Xpert) with a positive diagnostic test.

In sensitivity analyses, we assessed diagnostic accuracy using two other reference standards of combined culture or Xpert, and combined culture or Xpert among datasets that collected sputum for culture. We did not explore heterogeneity with meta-regression or assess for publication bias since few studies were included in each analysis. All meta-analyses were done using lme4, altmeta, meta, metafor, and mada packages in R (version 3.6.1).

This systematic review has been registered on PROSPERO (CRD42020155895).

### Role of the funding source

The funder (WHO) had a role in study design; data collection, analysis, and interpretation; and writing the report.

## Results

**Figure 1:**
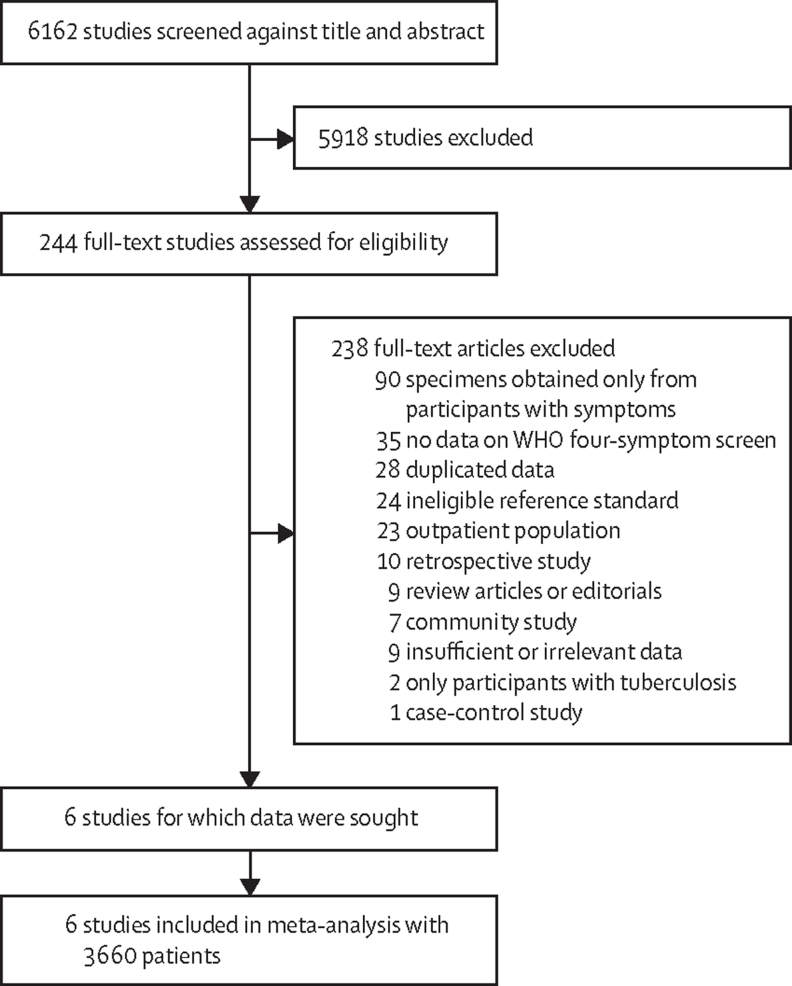
Study selection

Of 6162 publications found, six were eligible, and individual participant data were obtained for all six studies (n=3660; figure 1).^4^, ^14^, ^27^, ^28^, ^29^, ^30^ The characteristics of included studies are shown in the appendix (p 4). The included studies collected data from 2012 to 2017. All studies recruited inpatients from medical wards (one study recruited from an infectious disease ward). Five studies were done in sub-Saharan Africa and one in Asia. Studies systematically collected sputum for culture (four studies), sputum for Xpert (six studies), and urine for Xpert (two studies). We judged risk of bias for six studies that contributed to the proportion meta-analysis (appendix p 5). For the response rate domain, risk of bias was judged to be high for two studies that had a response rate of less than 80%. We judged risk of bias for four studies that contributed to the diagnostic meta-analysis with culture as reference standard (appendix p 6). For the reference test domain, risk of bias was judged to be high for three studies that did not obtain extrapulmonary samples for testing. The appendix (p 7) shows missing data by study. In three studies that did not exclude participants who could not produce sputum samples,^4^, ^14^, ^29^ sputum Xpert was missing for 35–54% of participants, mostly because inpatients were unable to produce a sputum sample, whereas urine Xpert was missing for 2% or less participants.

Participant characteristics overall are shown in table 1 and by study in the appendix (pp 8, 9). The median age of participants was 37 (IQR 31–45) years, 2104 (58%) of 3659 participants were women, and 2445 (67%) of 3642 participants were receiving ART. The median CD4 count was 205 (IQR 66–408) cells per μL. We did not collect data on ethnicity.

**Table 1:** Summary of main characteristics of participants

	**Number (%) or median (IQR)**	**Participants with data available for variable**
**Demographics**		
Age, years	37 (31–45)	3660
Female participants	2104 (58%)	3659
Male participants	1555 (42%)	3659
**HIV history**		
On ART	2445 (67%)	3642
CD4 count, cells per μL	205 (66–408)	3479
CD4 count ≤200 cells per μL	1709 (49%)	3479
**Clinical characteristics**		
History of tuberculosis diagnosis	902 (28%)	3268
W4SS positive*	3306 (90%)	3658
Cough	1945 (53%)	3655
Fever	1969 (54%)	3652
Weight loss	2638 (72%)	3651
Night sweats	1490 (41%)	3652
Cough for ≥2 weeks	765 (24%)	3172
Lymphadenopathy	58 (11%)	508
**Tuberculosis diagnostic tests**		
Total Xpert positive†	401 (14%)	2957
Total culture positive†	157 (23%)	674
**Imaging and laboratory tests**		
Chest X-ray, abnormal	130 (59%)	220
BMI, kg/m^2^	20 (18–24)	2966
CRP concentration, mg/L	75 (18–157)	400
CRP concentration ≥10 mg/L	334 (84%)	400
Haemoglobin concentration, g/dL	10 (8–12)	3481
Haemoglobin concentration <10 g/dL	1574 (45%)	3481

3660 participants were included in the study. ART=antiretroviral therapy. BMI=body-mass index. CRP=C-reactive protein. W4SS=WHO four-symptom screen. Xpert=Xpert MTB/RIF.

* W4SS is defined as one or more of the following symptoms: current cough, fever, night sweats, or weight loss.

† Sputum or non-sputum result.

**Figure 2:**
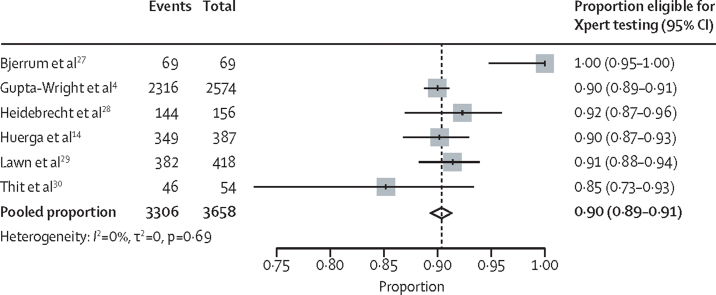
Random-effects meta-analysis of proportion of HIV-positive inpatients with positive WHO four-symptom screen (ie, proportion eligible for Xpert according to WHO algorithm) Xpert=Xpert MTB/RIF.

Among the four studies that collected sputum for culture, the pooled tuberculosis prevalence was 20% (95% CI 13–28; n=674) with culture as a reference standard and 25% (18–33; n=699) with culture or Xpert as a reference standard. Among six studies, the pooled proportion of inpatients with a positive W4SS (ie, inpatients eligible for Xpert testing according to the WHO algorithm) was 90% (89–91; n=3658); proportion estimates for individual studies ranged from 85% to 100% (figure 2).

**Figure 3:**
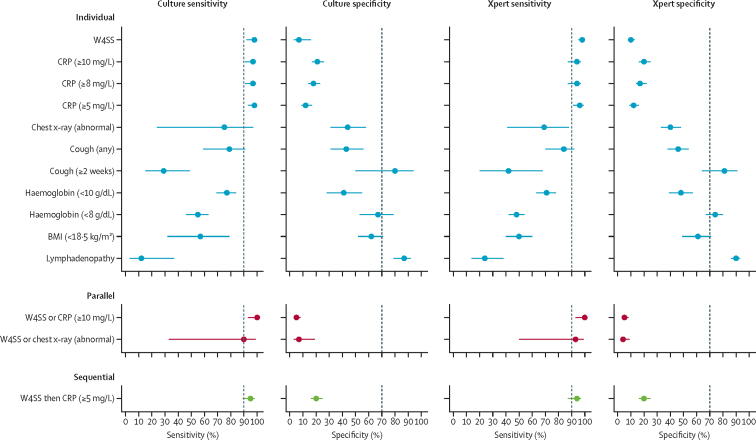
Pooled sensitivity and specificity along with 95% CIs for each screening test or strategy for the detection of tuberculosis using reference standards of culture or Xpert For parallel strategies, two screening tests are offered at the same time. For sequential strategies, a second screening test is offered only if the first screening test is positive. Dashed lines indicate WHO's minimum requirements for a tuberculosis screening test (90% sensitivity and 70% specificity). BMI=body-mass index. CRP=C-reactive protein. W4SS=WHO four-symptom screen. Xpert=Xpert MTB/RIF.

Plots of sensitivity and specificity for each screening test or strategy are shown in figure 3. Indirect comparisons are shown in table 2. For individual tests, the sensitivities of W4SS and CRP (≥5 mg/L) were highest, but the specificities were low. Cough (≥2 weeks), haemoglobin concentration (<8 g/dL), body-mass index (<18·5 kg/m^2^), and lymphadenopathy had moderate to high specificities, but low sensitivities, making them unsuitable to be explored as screening tests. Data on chest x-ray was sparse. In strategies that combined W4SS with CRP concentration, sensitivities were high, but specificities were low.

**Table 2:** Indirect comparisons of the diagnostic accuracy (pooled sensitivity and specificity) for each screening test or strategy for the detection of tuberculosis using reference standards of culture or Xpert

	**Culture**				Xpert*			
	Number of studies	Number of participants	Sensitivity (95% CI)	Specificity (95% CI)	Number of studies	Number of participants	Sensitivity (95% CI)	Specificity (95% CI)
**Screening test or strategy**								
W4SS	4	672	98% (92–99)	7% (3–16)	6	2176	98% (95–99)	10% (8–13)
CRP concentration ≥10 mg/L	1	400	97% (91–99)	21% (17–26)	1	395	94% (87–97)	20% (16–25)
CRP concentration ≥8 mg/L	1	400	97% (91–99)	18% (14–23)	1	395	94% (87–97)	17% (14–22)
CRP concentration ≥5 mg/L	1	400	98% (93–100)	12% (9–17)	1	395	96% (91–99)	12% (9–16)
Chest x-ray, abnormal	1	52	75% (24–97)	44% (31–58)	2	176	69% (41–88)	40% (33–48)
Cough, any	4	669	79% (59–91)	43% (31–56)	6	2173	84% (70–92)	46% (38–54)
Cough ≥2 weeks	3	608	29% (15–49)	80% (50–94)	4	1860	42% (20–68)	81% (64–91)
Haemoglobin concentration <10 g/dL	3	527	77% (69–84)	41% (28–55)	5	2015	71% (63–78)	48% (39–57)
Haemoglobin concentration <8 g/dL	3	527	55% (46–63)	67% (53–79)	5	2015	48% (42–54)	74% (67–80)
BMI <18·5 kg/m^2^	2	112	57% (32–79)	62% (52–71)	4	1553	50% (40–60)	61% (49–71)
Lymphadenopathy	2	123	12% (3–37)	87% (79–92)	3	337	24% (14–38)	90% (86–93)
W4SS or CRP ≥10 mg/L	1	399	100% (93–100)	5% (3–8)	1	394	100% (93–100)	5% (3–8)
W4SS or chest x-ray, abnormal	1	52	90% (33–99)	7% (3–19)	2	176	93% (50–99)	4% (2–9)
W4SS then CRP ≥5 mg/L	1	399	95% (89–98)	20% (16–25)	1	394	94% (87–97)	20% (16–25)
**Algorithm**								
WHO Xpert algorithm†‡	4	637	76% (67–84)	93% (88–96)	..	..	..	..
Xpert alone‡§	4	639	78% (69–85)	93% (87–96)	..	..	..	..

For parallel strategies, two screening tests are offered at the same time. For sequential strategies, a second screening test is offered only if the first screening test is positive. BMI=body-mass index. CRP=C-reactive protein. W4SS=WHO four-symptom screen. Xpert=Xpert MTB/RIF.

* In one study by Gupta-Wright and colleagues,^4^ only the intervention group was included since sputum Xpert and urine Xpert were available, whereas in the standard of care group, urine Xpert was unavailable and sputum Xpert was only available for 779 (61%) of 1287 participants.

† According to WHO Xpert algorithm, Xpert testing is advised if an inpatient has a positive W4SS (defined as one or more of the following symptoms: current cough, fever, night sweats, or weight loss).

‡ Accuracy measures for entire algorithm using sputum or urine Xpert result (or both); alternative algorithms are W4SS then single sputum Xpert (4 studies, 375 participants, sensitivity 78% [57–91], and specificity 97% [94–99]), single sputum Xpert alone (4 studies, 375 participants, sensitivity 78% [55–91], and specificity 97% [93–99]), and urine Xpert alone (1 study, 411 participants, sensitivity 59% [50–68], and specificity 91% [88–94]).

§ For Xpert alone, the comparator is the WHO Xpert algorithm.

Direct comparisons of individual tests were mostly similar to indirect comparisons (appendix pp 10, 11). Forest plots and summary receiver operating characteristics curves are provided in the appendix (pp 19–40). The appendix (pp 12–16) shows how estimates for each test or strategy affect a hypothetical cohort of 1000 HIV-positive inpatients at different tuberculosis prevalences. No individual test offered an optimal trade-off between tuberculosis cases missed and Xpert tests required (appendix p 41). In sensitivity analyses using alternative reference standards, results were largely similar to the main analyses (appendix p 17).

The sensitivity of the WHO Xpert algorithm (W4SS followed by Xpert) was 76% (95% CI 67–84) and specificity was 93% (88–96; n=637; table 2). The diagnostic accuracy of Xpert for all was similar to the WHO Xpert algorithm—sensitivity was 78% (69–85) and specificity was 93% (87–96; n=639). In a hypothetical cohort of 1000 HIV-positive inpatients at 20% tuberculosis prevalence, the WHO Xpert algorithm would result in 940 Xpert tests, but miss 48 tuberculosis cases; Xpert for all 1000 HIV-positive inpatients would miss 44 tuberculosis cases (appendix pp 12–14).

The appendix (p 18) shows diagnostic yield using different diagnostic tests and sample types. In one cohort that collected sputum and non-sputum samples for Xpert and culture,^29^ sputum Xpert diagnosed only 57 (41%) of all 139 tuberculosis cases (195 [46%] of 420 inpatients were unable to produce a sputum sample), whereas combined concentrated and unconcentrated urine Xpert diagnosed 89 (64%) of all 139 cases; concentrating urine increased diagnostic yield over not concentrating urine, from 42% to 59%. Sputum Xpert combined with urine Xpert diagnosed 116 (83%) of all 139 cases in the same cohort. In one cohort that collected sputum Xpert and concentrated urine Xpert, sputum Xpert diagnosed 85 (70%) of 122 tuberculosis cases and urine Xpert diagnosed 74 (61%) of 122 cases.^4^ Across all studies, Xpert was positive in six (2%) of 251 inpatients who had available Xpert results but were ineligible for Xpert testing according to the WHO Xpert algorithm.

## Discussion

In this individual participant data meta-analysis, we found that almost all HIV-positive inpatients in high-burden settings were eligible for Xpert testing using the WHO algorithm. W4SS and CRP concentration (≥5 mg/L) had the highest sensitivities of all screening tests evaluated to guide diagnostic testing, but specificities were low. Other screening tests had low sensitivities or wide 95% CIs. The WHO screening and diagnostic algorithm (ie, W4SS followed by Xpert) had a sensitivity of 76%; Xpert for all inpatients had similar sensitivity (78%). On the basis of these findings, WHO has made a strong recommendation to do molecular rapid diagnostic testing in all HIV-positive inpatients in high-burden settings (>10% tuberculosis prevalence).

We found that all screening tests and strategies to guide additional diagnostic testing fell short of WHO-defined minimum thresholds (90% sensitivity and 70% specificity) in this population.^13^ The specificity of W4SS in our study was only 7–10%, which is substantially lower than its specificity among outpatients on ART (71%) and not on ART (37%).^11^ The low specificity of CRP concentration for tuberculosis in this cohort is consistent with findings from other cohorts of symptomatic HIV-positive inpatients.^31^, ^32^ By contrast, CRP concentration has shown an improved specificity (67%) over W4SS in unselected outpatients not on ART.^11^ The low specificities of W4SS and CRP concentration are likely to be due to the high prevalences of other opportunistic diseases in patients without tuberculosis in this patient population. Both W4SS and CRP concentration met the minimum WHO sensitivity threshold of 90%. However, even at this high sensitivity, the W4SS and CRP concentration would miss roughly one in 50 tuberculosis cases. Given the high tuberculosis prevalence and the high mortality associated with missed diagnosis in inpatients in such settings, a small loss in sensitivity might be unacceptable.

Sputum Xpert alone had a low yield because many inpatients had difficulty producing sputum for testing. Urine-based Xpert testing might have an important role in diagnosing inpatients who are unable to produce sputum. We found that 35–54% of participants could not produce sputum for Xpert testing. In one cohort, sputum Xpert combined with urine Xpert diagnosed 83% of all cases, whereas sputum Xpert diagnosed only 41% of cases.^4^, ^29^ In the same cohort, urine Xpert had a higher yield than sputum Xpert,^29^ but the opposite was true in another cohort.^4^

There are several reasons to consider rapid diagnostic testing for tuberculosis with Xpert in all HIV-positive inpatients. First, since almost all inpatients with HIV met WHO eligibility requirements for Xpert testing, universal testing might reduce diagnostic complexity. Second, we found that Xpert was positive in 2% of HIV-positive inpatients who did not meet eligibility for testing. Third, in the real world, not all HIV-positive inpatients who qualify for Xpert testing might ultimately receive a test; for example, two of the included studies in this meta-analysis reported a positive W4SS in 90% or more HIV-positive inpatients, but clinicians identified only 38–64% as having possible tuberculosis after clinical assessment.^4^, ^14^ Fourth, since the W4SS is also used to assess eligibility for lateral flow urine lipoarabinomannan assay (LF-LAM) in HIV-positive inpatients, both routine Xpert and LF-LAM diagnostic testing might also be considered in this population. For instance, the STAMP trial showed a reduction in all-cause mortality among unselected HIV-positive inpatients when routine LF-LAM and urine Xpert were done in addition to routine sputum Xpert.^4^ Combined use of Xpert and LF-LAM has also been shown to improve diagnostic yield over either test alone in tuberculosis bloodstream infection, which predicts mortality.^33^ By contrast, obtaining Xpert samples in all HIV-positive inpatients might have a negative effect on infection control, human resources, laboratory capacity, and cost. However, since almost all inpatients already qualify for Xpert testing using the WHO criteria, Xpert testing in all inpatients would have a small effect on costs.

Although our findings support universal Xpert testing, this strategy would still miss more than 20% of culture-positive cases. Thus, aside from LF-LAM, additional diagnostic approaches that incorporate clinical symptoms and signs, radiological tests (eg, chest x-ray and abdominal ultrasound), and laboratory tests (eg, haemoglobin concentration) still have an important role in inpatients with a negative Xpert test.^34^, ^35^, ^36^, ^37^ Newer technologies might also substantially close this diagnostic gap. For example, Xpert Ultra and Fujifilm SILVAMP TB-LAM (FujiLAM) have shown increased sensitivity compared with Xpert and current LF-LAM tests.^38^, ^39^, ^40^ In a recent systematic review, Xpert Ultra increased sensitivity over Xpert by 13% (88% *vs* 75%) in sputum samples from people living with HIV.^39^ However, Xpert Ultra's lower specificity might have implications for universal Xpert Ultra testing because inpatients without tuberculosis could be classified as having tuberculosis.

Our study has limitations. First, most data were acquired in sub-Saharan Africa; the generalisability of this study to other geographical regions and low tuberculosis prevalence settings is unclear. Second, although we obtained and included data for all published studies identified by our search, some screening tests had wide 95% CIs because of sparse data. This limitation highlights the need for additional diagnostic accuracy studies among HIV-positive inpatients irrespective of tuberculosis signs and symptoms. Furthermore, no study evaluated Xpert Ultra and we did not assess other molecular WHO-recommended rapid diagnostic tests.^41^ Third, some studies only included participants able to produce sputum and did not collect extrapulmonary samples for culture or Xpert; two studies also did not collect culture samples. Since inpatients often present with extrapulmonary or disseminated tuberculosis and produce paucibacillary sputum samples, the reference standard in these studies might have introduced bias, underestimating the specificity and overestimating the sensitivity of existing algorithms. However, our results were consistent across several reference standards: culture, combinations of culture and Xpert, and Xpert (which is the currently recommended confirmatory test). Furthermore, estimates of the proportion of inpatients eligible for Xpert were based on data with higher methodological quality, since these analyses did not require a reference standard. Fourth, the small number of included studies precluded investigation of heterogeneity. Fifth, tuberculosis prevalence estimates in this Article are likely to be underestimates because of the limitations of our reference standard. Sixth, disseminated disease is more common at low CD4 counts, but we did not do analyses by CD4 count. However, HIV-positive inpatients typically present with advanced immunosuppression, and disseminated disease is not uncommon at higher CD4 counts in this population.^29^ Finally, our calculations for a hypothetical cohort should be treated with caution because they were based on diagnostic accuracy results derived from few participants, some of whom had an imperfect reference standard done.

In conclusion, our findings have informed the 2021 WHO recommendation to do molecular rapid diagnostic testing (eg, with Xpert) in all HIV-positive inpatients in high-burden settings (>10% tuberculosis prevalence). More accurate initial screening tests to guide additional diagnostic testing in HIV-positive inpatients need to be developed since current screening tests have suboptimal accuracy and hospitals in resource-limited settings might be unable to do systematic diagnostic testing in all HIV-positive inpatients. Although routine molecular rapid diagnostic testing might reduce the current diagnostic gap, a negative result still does not rule out tuberculosis. Xpert Ultra could additionally bridge the diagnostic gap and requires evaluation in unselected HIV-positive inpatients.

## Data sharing

The aggregate datasets are available on request. The study investigators of the original studies retain ownership of their data. Any requests for access to individual participant data should be made directly to the study investigators of the original studies.

## Declaration of interests

We declare no competing interests.
