# Supplementary material for: Tuberculosis screening among HIV-positive inpatients: a systematic review and individual participant data meta-analysis
Source: Lancet HIV. 2022 Mar 23;9(4):e233–41. doi: 10.1016/S2352-3018(22)00002-9 (PMC8964502; doi:10.1016/S2352-3018(22)00002-9)
Supplement: Supplementary appendix [file mmc1.pdf]

THE LANCET
HIV

Supplementary appendix

This appendix formed part of the original submission and has been peer reviewed. We post it as supplied by the authors.

Supplement to: Dhana A, Hamada Y, Kengne A P, et al. Tuberculosis screening among HIV-positive inpatients: a systematic review and individual participant data meta-analysis. *Lancet HIV* 2022; published online March 23. https://doi.org/10.1016/S2352-3018(22)00002-9
.

Supplementary Figures and Tables

15/12/2021

Table of Contents

Table S1 - Search terms 2
Table S2 - Variables sought 3
Table S3 - Study-level characteristics 4
Table S4 - Risk of bias results of studies that assessed proportion of HIV-positive inpatients eligible for Xpert 5
Table S5 - Risk of bias and applicability results on the QUADAS-2 criteria tool among studies with culture-based reference standard* 6
Table S6 - Percentage of missing data for each variable by study†§ 7
Table S7 - Summary of main characteristics for participants overall and by each study 8
Table S8 - Direct comparisons of the diagnostic accuracy (pooled sensitivity and specificity) between each screening test/strategy and WHO four-symptom screen for the detection of tuberculosis 10
Table S9 - Translation of pooled sensitivity and specificity estimates of different screening tests/strategies and diagnostic algorithms to a population of 1000 persons 12
Table S10 - Sensitivity analyses of diagnostic accuracy (pooled sensitivity and specificity) for each screening test/strategy for the detection of tuberculosis using an alternative reference standard of culture or Xpert* 17
Table S11 - Diagnostic yield of different Xpert tests and sample types as a proportion of total microbiologically confirmed tuberculosis cases† 18
Figure S1 - Forest plots of sensitivity and specificity estimates for each screening test/strategy (C-reactive protein >=8 mg/L omitted) 19
Figure S2 - Summary receiver operating characteristics curves for each screening test/strategy (for tests/strategies with >=4 studies available) 38
Figure S3 - Plot comparing number of tuberculosis cases missed with number of Xpert tests required for different tuberculosis screening tests when screening a population of 1000 persons† 42
References 43
PRISMA-IPD checklist 44

Table S1 - Search terms

Database	Search terms
Pubmed	
#1.	"HIV Infections" [MeSH] OR "HIV"[MeSH] OR "hiv"[tw] OR hiv infect*[tw] OR "human immunodeficiency virus"[tw] OR "human immunedeficiency virus"[tw] OR "human immuno-deficiency virus"[tw] OR "human immune-deficiency virus"[tw] OR ((human immun*) AND ("deficiency virus"[tw])) OR "acquired immunodeficiency syndrome"[tw] OR "acquired immunedeficiency syndrome"[tw] OR "acquired immuno-deficiency syndrome"[tw] OR "acquired immune-deficiency syndrome"[tw] OR ((acquired immun*) AND ("deficiency syndrome"[tw]))
#2.	"Tuberculosis"[Mesh] OR tuberculosis [TW] OR "Mycobacterium tuberculosis"[Mesh] OR TB [Ti]
#3	Screening* OR algorithm* OR "case finding" [TIAB] OR "case findings" [TIAB] OR sensitivit* OR specificit* OR predictor* OR "Sensitivity and Specificity"[MeSH Terms] OR "Tuberculosis/diagnosis"[Mesh] OR "Mass Screening"[Mesh:NoExp]
#4.	("animals"[MeSH Terms] NOT ("humans"[MeSH Terms] AND "animals"[MeSH Terms])) OR case reports[Publication Type]
#5	#1 AND #2 AND #3 NOT #4
	Limit: publication date from 2011/01/01
Embase	
#1	'human immunodeficiency virus infection'/exp OR 'human immunodeficiency virus'/exp OR 'hiv':ti,ab OR 'human immunodeficiency virus':ti,ab OR 'human immuno-deficiency virus':ti,ab OR 'human immunedeficiency virus':ti,ab OR 'human immune-deficiency virus':ti,ab OR 'acquired immune-deficiency syndrome':ti,ab OR 'acquired immunedeficiency syndrome':ti,ab OR 'acquired immunodeficiency syndrome':ti,ab OR 'acquired immuno-deficiency syndrome':ti,ab
#2	'tuberculosis'/exp OR 'tuberculosis':ab,ti OR 'TB':ti OR 'Mycobacterium tuberculosis'/exp
#3	'Screen':ti,ab OR 'Screening':ti,ab OR 'algorithm':ti,ab OR 'case finding':ti,ab OR 'case findings':ti,ab OR sensitivit*:ti,ab OR specificit*:ti,ab OR predictor*:ti,ab OR 'sensitivity and specificity'/exp OR 'case finding'/exp OR 'Mass Screening'/exp OR 'screening'/exp
#4	([animals]/lim NOT [humans]/lim)
#5	#1 AND #2 AND #3 NOT #4 AND [2011-]/py
Cochrane	
#1.	"HIV Infections" [MeSH] OR "HIV"[MeSH] OR hiv OR hiv infect* OR "human immunodeficiency virus" OR "human immunedeficiency virus"OR "human immuno-deficiency virus" OR "human immune-deficiency virus" OR ((human immun*) AND ("deficiency virus")) OR "acquired immunodeficiency syndrome" OR "acquired immunedeficiency syndrome" OR "acquired immuno-deficiency syndrome" OR "acquired immune-deficiency syndrome" OR ((acquired immun*) AND ("deficiency syndrome"))
#2.	"Tuberculosis"[Mesh] OR tuberculosis OR "Mycobacterium tuberculosis"[Mesh]
#3	Screening* OR algorithm* OR "case finding" OR "case findings" OR sensitivit* OR specificit* OR predictor* OR "Sensitivity and Specificity"[MeSH Terms] OR "Tuberculosis/diagnosis"[Mesh] OR "Mass Screening"[Mesh:NoExp]
#4.	("animals"[MeSH Terms] NOT ("humans"[MeSH Terms] AND "animals"[MeSH Terms])) OR case reports[Mesh]
#5	#1 AND #2 AND #3 NOT #4
	Limit: publication year from 2011-

Table S2 - Variables sought

Variable	Description
country	country where the study took place, or if multisite, country individual patient was recruited from
clinical setting	from {inpatient, outpatient, other, NA}
age	patient’s age in years
sex	patient’s sex {female, male, NA}
hiv status	from {positive, negative, NA}
art status	from {on art, not on art, NA}
history of tuberculosis	from {history of tuberculosis, no history of tuberculosis, NA}
current smoking status	from {currently smoking, not currently smoking, NA}
pregnancy status	from {pregnant, not pregnant, NA}
tuberculosis treatment status	from {currently on tuberculosis treatment, not currently on tuberculosis treatment, NA}
current ipt status	from {yes, no, NA}
current cough	from {yes, no, NA}
cough (more than 2 weeks)	from {yes, no, NA}
fever	from {yes, no, NA}
weight loss	from {yes, no, NA}
night sweats	from {yes, no, NA}
w4ss	number of w4ss symptoms {0, 1, 2, 3, 4, NA}
body mass index	numerical value (weight/height^2)
lymphadenopathy	from {yes, no, NA}
cd4 count	numerical value (in cells/µL)
c-reactive protein level	numerical value (in mg/L)
haemoglobin	numerical value (in g/dl)
chest x-ray suggestive of tuberculosis	from {yes, no, NA}
chest x-ray abnormal	from {yes, no, NA}
sputum xpert result	{positive, negative, NA}, indeterminate = negative
sputum culture result	{positive, negative, NA}, contaminated culture = negative
non-sputum xpert result	{positive, negative, NA}, indeterminate = negative
non-sputum culture result	{positive, negative, NA}, contaminated culture = negative

Definition of abbreviations: ART = antiretroviral therapy, IPT = Isoniazid preventive therapy, W4SS = WHO four-symptom screen

Table S3 - Study-level characteristics

Author, year	Country	Study period	Study population	Study setting	Exclusion criteria	Sputum culture	Sputum Xpert	Liquid or solid culture	Non-sputum culture/Xpert
Bjerrum, 2015[1]	Ghana	2013-2014	ART-naïve inpatient PLHIV aged ≥18 years with WHO stage 3/4 or CD4 cell count ≤350 per µL or pregnant admitted to the Fevers Unit (infectious diseases ward)	1 hospital	On ATT for >2 days in 3 months before admission or unable to produce sputum or urine samples	1 spot and 1 early morning samples	1 or 2 samples	Both	No
Gupta-Wright, 2018[2]	South Africa and Malawi	2015-2017	Inpatient PLHIV admitted to medical wards	2 hospitals	On ATT, treated for TB in previous 12 months, IPT in previous 6 months, admitted to hospital for >48 hours at time of screening	-	1 spot sample, induced if physician requested at 1 site	-	Urine Xpert in intervention group
Heidebrecht, 2016[3]	South Africa	2013-2013	Inpatient PLHIV admitted to medical wards	1 hospital	≥3 doses of ATT	1 spot sample, induced if physician requested	1 spot sample, induced if physician requested	Both	No
Huerga, 2021[4]	Malawi	2015-2017	Inpatient PLHIV aged ≥15 years admitted to medical wards	1 hospital	On ATT	-	1 spot sample	-	-
Lawn, 2015[5]	South Africa	2012-2013	Inpatient PLHIV aged ≥18 years admitted to medical wards	1 district hospital	Current TB diagnosis and/or were receiving ATT at the time of admission	1 spot and 1 induced samples, 2 induced if necessary, if too unwell for induction then 2 spot samples, additional samples according to medical team	1 spot and 1 induced sample, 2 induced if necessary, if too unwell for induction then 2 spot samples, additional samples according to medical team	Liquid	Blood culture, urine Xpert (fresh and frozen), other samples if clinically indicated
Thit, 2017[6]	Myanmar	2015-2015	Inpatient PLHIV admitted to medical wards	1 tertiary hospital	-	1 spot sample, induced if unable to expectorate	1 spot sample, induced if unable to expectorate	Solid	No

Definition of abbreviations: ART = antiretroviral therapy, ATT = anti-tuberculosis treatment, PLHIV = people living with HIV, TB = tuberculosis

Table S4 - Risk of bias results of studies that assessed proportion of HIV-positive inpatients eligible for Xpert

Domain	Gupta-Wright, 2018	Huerga, 2021	Lawn, 2015	Heidebrecht, 2016	Bjerrum, 2015	Thit, 2017
**1. Was the sample frame appropriate to address the target population?¶**	Yes	Yes	Yes	Yes	No¶¶	Yes
**2. Were study participants recruited in an appropriate way?§**	Yes	Yes	Yes	Yes	Yes	Yes
**3. Were the study subjects and setting described in detail?***	Yes	Yes	Yes	Yes	Yes	Yes
**4. Were valid methods used for the identification of eligibility criteria?#**	Yes	Yes	Yes	Yes	Yes	Yes
**5. Was the response rate adequate (>80%)?**	No	Yes	Yes	No	Yes	Yes

¶The sample frame was considered inappropriate if a certain group was used and the results were then inferred to the target population

¶¶For Bjerrum et al (2015), study inclusion criteria were ART naïve, WHO stage 3/4 or CD4 count <=350 per μL or pregnant

§Was recruitment conducted using a consecutive or random sample?

*Was the study sample described in sufficient detail so that other researchers can determine if it is comparable to the population of interest to them? For example, did the study report age, gender, ART status, and CD4 count?

#Were eligibility items (i.e., WHO four-symptom screen) assessed based on existing definitions or diagnostic criteria?

Definition of abbreviations: ART = antiretroviral therapy

Table S5 - Risk of bias and applicability results on the QUADAS-2 criteria tool among studies with culture-based reference standard*

Domain	Bjerrum, 2015	Heidebrecht, 2016	Lawn, 2015	Thit, 2017
**Patient selection (Risk of Bias)¶**	Low	Low	Low	Low
**Index test (Risk of Bias)¶¶**	Low	Low	Low	Low
**Reference test (Risk of Bias)§**	High	High	Low	High
**Flow and timing (Risk of Bias)#**	Low	Low	High	Low
**Patient selection (Applicability)†**	High	Low	Low	Low
**Index test (Applicability)†**	Low	Low	Low	Low
**Reference test (Applicability)†**	Low	Low	Low	Low

*Assessment done for all index tests

¶Was a consecutive or random sample of patients enrolled? Did the study avoid inappropriate exclusions?

¶¶Were the index test results interpreted without knowledge of the results of the reference standard?

§Is the reference standard likely to correctly classify the target condition? For example, were both pulmonary and extrapulmonary samples obtained? Were the reference standard results interpreted without knowledge of the results of the index test?

#Was there an appropriate interval between index test(s) and reference standard? Did all patients receive a reference standard? Did all patients receive the same reference standard? Were all patients included in the analysis?

†Are there concerns that the included patients (patient selection), index test, or target condition (reference standard) do not match the review question?

Table S6 - Percentage of missing data for each variable by study†§

Variable†	Bjerrum	Gupta-Wright Intervention††	Gupta-Wright Control††	Heidebrecht	Huerga	Lawn	Thit
**Clinical setting**	0	0	0	0	0	0	0
**Age**	0	0	0	0	0	0	0
**Sex**	0	0	0	1	0	0	0
**ART status**	0	0	0	2	4	0	0
**History of tuberculosis**	0	0	0	2	100	0	0
**W4SS***	0	0	0	0	0	0	0
Cough	1	0	0	1	0	1	0
Fever	4	0	0	0	1	1	0
Weight loss	1	0	0	0	1	0	0
Night sweats	0	0	0	2	0	1	0
**Cough >=2 weeks**	1	1	1	6	100	1	100
**BMI**	14	0	0	100	28	100	2
**Lymphadenopathy**	0	100	100	100	1	100	0
**CD4 count**	10	0	1	100	2	0	0
**CRP**	100	100	100	100	100	5	100
**Haemoglobin**	6	0	0	100	1	1	11
**CXR (abnormal)****	100	100	100	100	57	100	4
**Sputum Xpert*****	28	35	39	6	39	54	0
**Non-sputum Xpert**	100	1	100	100	100	2	100
**Total Xpert*****	28	1	39	6	39	1	0
**Sputum culture**	0	100	100	16	100	50	0
**Non-sputum culture**	100	100	100	100	100	0	100
**Total culture**	0	100	100	16	100	0	0
**Total (culture or Xpert)**	0	1	39	0	39	0	0

†<5% missing (green), 5-95% missing (yellow), and >95% missing (red)

§Some datasets received in which some participants with missing data were already excluded

††Study by Gupta-Wright involved an intervention arm (systematically performed urine Xpert and sputum Xpert) and control arm (systematically performed sputum Xpert only)

*Regarded as missing only if a subject had all four symptoms missing

**Study by Huerga et al has a high missing value for CXR (abnormal) because the study site at times had a lack of water and technicians to perform chest x-ray

***Study by Bjerrum et al has a high missing value for Xpert because Xpert only became available after study enrollment began

Definition of abbreviations: ART = antiretroviral therapy, BMI = body mass index, CRP = C-reactive protein, CXR = chest X-ray, Hb = haemoglobin, W4SS = WHO four-symptom screen

Table S7 - Summary of main characteristics for participants overall and by each study

Variable†	All	Bjerrum	Gupta-Wright Intervention††	Gupta-Wright Control††	Heidebrecht	Huerga	Lawn	Thit
**Frequency**	3660 (100)	69 (2)	1287 (35)	1287 (35)	156 (4)	387 (11)	420 (11)	54 (1)
**Age (years)**	37 (31-45)	37 (32-43)	38 (31-46)	38 (31-46)	36 (28-44)	38 (32-45)	36 (29-42)	33 (30-44)
**N**	3660	69	1287	1287	156	387	420	54
**CD4 count (cells/μL)**	205 (66-408)	41 (12-115)	231 (78-438)	222 (80-436)	-	173 (51-370)	150 (56-312)	97 (42-264)
**N**	3479	62	1286	1279	-	380	418	54
**CD4 <=200 cells/μL**	1709 (49)	53 (85)	572 (44)	592 (46)	-	205 (54)	252 (60)	35 (65)
**N**	3479	62	1286	1279	-	380	418	54
**Female**	2104 (58)	33 (48)	727 (56)	734 (57)	112 (72)	216 (56)	255 (61)	27 (50)
**N**	3659	69	1287	1287	155	387	420	54
**On ART**	2445 (67)	0 (0)	926 (72)	935 (73)	82 (54)	305 (82)	175 (42)	22 (41)
**N**	3642	69	1287	1287	153	372	420	54
**History of TB**	902 (28)	5 (7)	335 (26)	309 (24)	46 (30)	-	190 (45)	17 (31)
**N**	3268	69	1287	1287	153	-	418	54
**Current Smoker**	293 (11)	1 (2)	151 (12)	128 (10)	-	-	-	13 (24)
**N**	2693	65	1287	1287	-	-	-	54
**W4SS***	3306 (90)	69 (100)	1152 (90)	1164 (90)	144 (92)	349 (90)	382 (91)	46 (85)
**N**	3658	69	1287	1287	156	387	418	54
**Cough**	1945 (53)	48 (71)	651 (51)	681 (53)	111 (72)	230 (59)	199 (48)	25 (46)
**N**	3655	68	1287	1287	155	387	417	54
**Fever**	1969 (54)	46 (70)	753 (59)	747 (58)	98 (63)	228 (59)	62 (15)	35 (65)
**N**	3652	66	1287	1287	156	385	417	54
**Weight loss**	2638 (72)	65 (96)	906 (70)	875 (68)	117 (75)	277 (73)	356 (85)	42 (78)
**N**	3651	68	1287	1286	156	382	418	54
**Night sweats**	1490 (41)	29 (42)	497 (39)	540 (42)	76 (50)	154 (40)	171 (41)	23 (43)
**N**	3652	69	1287	1286	153	386	417	54
**Cough >= 2 weeks**	765 (24)	35 (51)	342 (27)	321 (25)	34 (23)	-	33 (8)	-
**N**	3172	68	1271	1270	147	-	416	-
**Lymphadenopathy**	58 (11)	8 (12)	-	-	-	42 (11)	-	8 (15)
**N**	508	69	-	-	-	385	-	54
**CXR (abnormal)**	130 (59)	-	-	-	-	100 (60)	-	30 (58)
**N**	220	-	-	-	-	168	-	52
**Sputum Xpert +**	305 (13)	9 (18)	85 (10)	82 (11)	35 (24)	33 (14)	57 (29)	4 (7)
**N**	2291	50	832	779	146	235	195	54
**Non-sputum Xpert +**	163 (10)	-	74 (6)	-	-	-	89 (22)	-
**N**	1681	-	1270	-	-	-	411	-
**Total Xpert +§**	401 (14)	9 (18)	122 (10)	82 (11)	35 (24)	33 (14)	116 (28)	4 (7)
**N**	2957	50	1279	779	146	235	414	54
**Sputum culture +**	106 (23)	13 (19)	-	-	31 (24)	-	58 (28)	4 (7)
**N**	463	69	-	-	131	-	209	54
**Non-sputum culture +**	70 (17)	-	-	-	-	-	70 (17)	-
**N**	420	-	-	-	-	-	420	-
**Total culture +¶**	157 (23)	13 (19)	-	-	31 (24)	-	109 (26)	4 (7)
**N**	674	69	-	-	131	-	420	54
**Total Xpert & culture +**	439 (15)	15 (22)	122 (10)	82 (11)	41 (26)	33 (14)	139 (33)	7 (13)

Variable†	All	Bjerrum	Gupta-Wright Intervention††	Gupta-Wright Control††	Heidebrecht	Huerga	Lawn	Thit
**N**	2992	69	1279	779	156	235	420	54
**BMI (kg/m2)**	20 (18-24)	19 (17-21)	21 (18-24)	21 (18-24)	-	18 (17-21)	-	20 (17-21)
**N**	2966	59	1287	1287	-	280	-	53
**CRP (mg/L)**	75 (18-157)	-	-	-	-	-	75 (18-157)	-
**N**	400	-	-	-	-	-	400	-
**CRP (>=10 mg/L)**	334 (84)	-	-	-	-	-	334 (84)	-
**N**	400	-	-	-	-	-	400	-
**Hb, Median (g/dL)**	10 (8-12)	7 (5-10)	11 (8-13)	11 (8-13)	-	9 (7-11)	10 (8-12)	9 (7-11)
**N**	3481	65	1284	1285	-	385	414	48
**Hb (<10 g/dL)**	1574 (45)	50 (77)	544 (42)	505 (39)	-	219 (57)	227 (55)	29 (60)
**N**	3481	65	1284	1285	-	385	414	48

†Data are count (%) or median (25th-75th percentiles)

††Study by Gupta-Wright involved an intervention arm (systematically collected urine Xpert and sputum Xpert) and control arm (systematically collected sputum Xpert only)

*W4SS defined as one or more of the following: current cough, fever, night sweats, or weight loss

§Sputum and/or non-sputum Xpert result

¶Sputum and/or non-sputum culture result

Definition of abbreviations: ART = antiretroviral therapy, BMI = body mass index, CRP = C-reactive protein, CXR = chest X-ray, Hb = haemoglobin, W4SS = WHO four-symptom screen

Table S8 - Direct comparisons of the diagnostic accuracy (pooled sensitivity and specificity) between each screening test/strategy and WHO four-symptom screen for the detection of tuberculosis

Table S8A - Direct comparisons of the diagnostic accuracy (pooled sensitivity and specificity) between each screening test/strategy and WHO four-symptom screen for the detection of tuberculosis in all participants using culture as reference standard†

	Index Test				W4SS			
	No of studies	N	Sensitivity (95% CI)	Specificity (95% CI)	No of studies	N	Sensitivity (95% CI)	Specificity (95% CI)
**CRP (>=10 mg/L)**	1	399	97 (91-99)	21 (17-26)	1	399	97 (91-99)	10 (7-14)
**CRP (>=8 mg/L)**	1	399	97 (91-99)	18 (14-23)	1	399	97 (91-99)	10 (7-14)
**CRP (>=5 mg/L)**	1	399	98 (92-100)	12 (9-17)	1	399	97 (91-99)	10 (7-14)
**CXR (abnormal)**	1	52	75 (24-97)	44 (31-58)	1	52	90 (33-99)	17 (9-31)
**Cough (any)**	4	669	79 (58-91)	43 (31-57)	4	669	98 (93-100)	8 (4-14)
**Cough (>=2 weeks)**	3	608	28 (16-46)	80 (52-93)	3	608	98 (92-99)	5 (1-17)
**Hb (<10 g/dL)**	3	525	78 (70-84)	40 (26-57)	3	525	98 (93-99)	8 (4-17)
**Hb (<8 g/dL)**	3	525	55 (46-64)	67 (50-81)	3	525	98 (93-99)	8 (4-17)
**BMI (<18.5 kg/m²)§**	2	112	57 (32-79)	62 (44-78)	2	112	100 (85-100)	6 (2-18)
**Lymphadenopathy§**	2	123	12 (3-37)	87 (68-96)	2	123	100 (83-100)	6 (2-18)
**W4SS or CRP (>=10 mg/L)¶**	1	399	100 (93-100)	5 (3-8)	1	399	97 (91-99)	10 (7-14)
**W4SS or CXR (abnormal)¶**	1	52	90 (33-99)	7 (3-19)	1	52	90 (33-99)	17 (9-31)
**W4SS then CRP (>=5 mg/L)¶**	1	399	95 (89-98)	20 (16-25)	1	399	97 (91-99)	10 (7-14)

†Direct comparisons are based on all studies that evaluated both the W4SS and relevant screening test

§We computed binomial 95% CIs for W4SS sensitivity as all studies had 100% sensitivity

¶For parallel strategies, two screening tests are offered at the same time. For sequential strategies, a second screening test is offered only if the first screening test is positive

Definition of abbreviations: BMI = body mass index, CRP = C-reactive protein, CXR = chest X-ray, Hb = haemoglobin, W4SS = WHO four-symptom screen

Table S8B - Direct comparisons of the diagnostic accuracy (pooled sensitivity and specificity) between each screening test/strategy and WHO four-symptom screen for the detection of tuberculosis in all participants using Xpert as reference standard†

	Index Test				W4SS			
	No of studies	N	Sensitivity (95% CI)	Specificity (95% CI)	No of studies	N	Sensitivity (95% CI)	Specificity (95% CI)
**CRP (>=10 mg/L)**	1	394	94 (87-97)	20 (16-25)	1	394	97 (92-99)	11 (8-15)
**CRP (>=8 mg/L)**	1	394	94 (87-97)	17 (14-22)	1	394	97 (92-99)	11 (8-15)
**CRP (>=5 mg/L)**	1	394	96 (91-99)	12 (9-16)	1	394	97 (92-99)	11 (8-15)
**CXR (abnormal)**	2	176	69 (41-88)	40 (33-48)	2	176	92 (61-99)	9 (5-14)
**Cough (any)**	6	2,173	84 (70-92)	46 (39-54)	6	2,173	99 (96-100)	10 (7-13)
**Cough (>=2 weeks)**	4	1,860	42 (22-65)	81 (67-90)	4	1,860	99 (96-100)	8 (4-17)
**Hb (<10 g/dL)**	5	2,013	72 (64-79)	48 (40-57)	5	2,013	99 (97-100)	9 (7-13)
**Hb (<8 g/dL)**	5	2,013	49 (41-58)	73 (67-79)	5	2,013	99 (97-100)	10 (7-13)
**BMI (<18.5 kg/m²)§**	4	1,553	50 (42-57)	61 (49-71)	4	1,553	100 (98-100)	8 (5-13)
**Lymphadenopathy**	3	337	24 (14-38)	90 (86-93)	3	337	98 (86-100)	7 (4-10)
**W4SS or CRP (>=10 mg/L)¶**	1	394	100 (93-100)	5 (3-8)	1	394	97 (92-99)	11 (8-15)
**W4SS or CXR (abnormal)¶**	2	176	93 (54-99)	4 (2-9)	2	176	93 (54-99)	9 (5-14)
**W4SS then CRP (>=5 mg/L)¶**	1	394	94 (87-97)	20 (16-25)	1	394	97 (92-99)	11 (8-15)

†Direct comparisons are based on all studies that evaluated both the W4SS and relevant screening test

§Bivariate model did not converge; results from a univariate random-effects model. We computed binomial 95% CIs for W4SS sensitivity as all studies had 100% sensitivity

¶For parallel strategies, two screening tests are offered at the same time. For sequential strategies, a second screening test is offered only if the first screening test is positive

Definition of abbreviations: BMI = body mass index, CRP = C-reactive protein, CXR = chest X-ray, Hb = haemoglobin, W4SS = WHO four-symptom screen

Table S9 - Translation of pooled sensitivity and specificity estimates of different screening tests/strategies and diagnostic algorithms to a population of 1000 persons

Table S9A - Translation of pooled sensitivity and specificity estimates of different screening tests/strategies and diagnostic algorithms to a population of 1000 persons using culture as a reference standard§

		Outcome of screening§§							Outcome of screening then diagnosis§§						
**Test**	**Total TB**	**TP+FP‡**	**TP**	**FP**	**TN**	**FN**	**PPV**	**NPV**	**TP**	**TN**	**FP**	**FN**	**PPV**	**NPV**	**NNS**
	5% prevalence														
**W4SS**	50	932	49	883	67	1	5.3	98.5	-	-	-	-	-	-	-
**CRP (>=10 mg/L)**	50	799	48	750	200	2	6.1	99.3	-	-	-	-	-	-	-
**CRP (>=8 mg/L)**	50	828	48	779	171	2	5.9	99.1	-	-	-	-	-	-	-
**CRP (>=5 mg/L)**	50	885	49	836	114	1	5.5	99.1	-	-	-	-	-	-	-
**CXR (abnormal)**	50	570	38	532	418	12	6.6	97.1	-	-	-	-	-	-	-
**Cough (any)**	50	581	40	542	408	10	6.8	97.5	-	-	-	-	-	-	-
**Cough (>=2 weeks)**	50	204	14	190	760	36	7.1	95.5	-	-	-	-	-	-	-
**Hb (<10 g/dL)**	50	599	38	560	390	12	6.4	97.1	-	-	-	-	-	-	-
**Hb (<8 g/dL)**	50	341	28	313	637	22	8.1	96.6	-	-	-	-	-	-	-
**BMI (<18.5 kg/m²)**	50	390	29	361	589	21	7.3	96.5	-	-	-	-	-	-	-
**Lymphadenopathy**	50	130	6	124	826	44	4.6	94.9	-	-	-	-	-	-	-
**W4SS or CRP (>=10 mg/L)¶**	50	952	50	902	48	0	5.2	100	-	-	-	-	-	-	-
**W4SS or CXR (abnormal)¶**	50	928	45	883	67	5	4.8	93	-	-	-	-	-	-	-
**W4SS then CRP (>=5 mg/L)¶**	50	808	48	760	190	2	5.9	98.7	-	-	-	-	-	-	-
**WHO Xpert algorithm*†**	50	-	-	-	-	-	-	-	38	884	66	12	36.4	98.7	26
**Xpert alone*†**	50	-	-	-	-	-	-	-	39	884	66	11	37	98.8	26
	10% prevalence														
**W4SS**	100	935	98	837	63	2	10.5	96.9	-	-	-	-	-	-	-
**CRP (>=10 mg/L)**	100	808	97	711	189	3	12	98.4	-	-	-	-	-	-	-
**CRP (>=8 mg/L)**	100	835	97	738	162	3	11.6	98.2	-	-	-	-	-	-	-
**CRP (>=5 mg/L)**	100	890	98	792	108	2	11	98.2	-	-	-	-	-	-	-
**CXR (abnormal)**	100	579	75	504	396	25	13	94.1	-	-	-	-	-	-	-
**Cough (any)**	100	592	79	513	387	21	13.3	94.9	-	-	-	-	-	-	-
**Cough (>=2 weeks)**	100	209	29	180	720	71	13.9	91	-	-	-	-	-	-	-
**Hb (<10 g/dL)**	100	608	77	531	369	23	12.7	94.1	-	-	-	-	-	-	-
**Hb (<8 g/dL)**	100	352	55	297	603	45	15.6	93.1	-	-	-	-	-	-	-
**BMI (<18.5 kg/m²)**	100	399	57	342	558	43	14.3	92.8	-	-	-	-	-	-	-
**Lymphadenopathy**	100	129	12	117	783	88	9.3	89.9	-	-	-	-	-	-	-
**W4SS or CRP (>=10 mg/L)¶**	100	955	100	855	45	0	10.5	100	-	-	-	-	-	-	-
**W4SS or CXR (abnormal)¶**	100	927	90	837	63	10	9.7	86.3	-	-	-	-	-	-	-
**W4SS then CRP (>=5 mg/L)¶**	100	815	95	720	180	5	11.7	97.3	-	-	-	-	-	-	-

		Outcome of screening§§							Outcome of screening then diagnosis§§							
**Test**	**Total TB**	**TP+FP‡**	**TP**	**FP**	**TN**	**FN**	**PPV**	**NPV**	**TP**	**TN**	**FP**	**FN**	**PPV**	**NPV**	**NNS**
**WHO Xpert algorithm*†**	100	-	-	-	-	-	-	-	76	837	63	24	54.7	97.2	13
**Xpert alone*†**	100	-	-	-	-	-	-	-	78	837	63	22	55.3	97.4	13
	20% prevalence														
**W4SS**	200	940	196	744	56	4	20.9	93.3	-	-	-	-	-	-	-
**CRP (>=10 mg/L)**	200	826	194	632	168	6	23.5	96.6	-	-	-	-	-	-	-
**CRP (>=8 mg/L)**	200	850	194	656	144	6	22.8	96	-	-	-	-	-	-	-
**CRP (>=5 mg/L)**	200	900	196	704	96	4	21.8	96	-	-	-	-	-	-	-
**CXR (abnormal)**	200	598	150	448	352	50	25.1	87.6	-	-	-	-	-	-	-
**Cough (any)**	200	614	158	456	344	42	25.7	89.1	-	-	-	-	-	-	-
**Cough (>=2 weeks)**	200	218	58	160	640	142	26.6	81.8	-	-	-	-	-	-	-
**Hb (<10 g/dL)**	200	626	154	472	328	46	24.6	87.7	-	-	-	-	-	-	-
**Hb (<8 g/dL)**	200	374	110	264	536	90	29.4	85.6	-	-	-	-	-	-	-
**BMI (<18.5 kg/m²)**	200	418	114	304	496	86	27.3	85.2	-	-	-	-	-	-	-
**Lymphadenopathy**	200	128	24	104	696	176	18.8	79.8	-	-	-	-	-	-	-
**W4SS or CRP (>=10 mg/L)¶**	200	960	200	760	40	0	20.8	100	-	-	-	-	-	-	-
**W4SS or CXR (abnormal)¶**	200	924	180	744	56	20	19.5	73.7	-	-	-	-	-	-	-
**W4SS then CRP (>=5 mg/L)¶**	200	830	190	640	160	10	22.9	94.1	-	-	-	-	-	-	-
**WHO Xpert algorithm*†**	200	-	-	-	-	-	-	-	152	744	56	48	73.1	93.9	7
**Xpert alone*†**	200	-	-	-	-	-	-	-	156	744	56	44	73.6	94.4	6
	30% prevalence														
**W4SS**	300	945	294	651	49	6	31.1	89.1	-	-	-	-	-	-	-
**CRP (>=10 mg/L)**	300	844	291	553	147	9	34.5	94.2	-	-	-	-	-	-	-
**CRP (>=8 mg/L)**	300	865	291	574	126	9	33.6	93.3	-	-	-	-	-	-	-
**CRP (>=5 mg/L)**	300	910	294	616	84	6	32.3	93.3	-	-	-	-	-	-	-
**CXR (abnormal)**	300	617	225	392	308	75	36.5	80.4	-	-	-	-	-	-	-
**Cough (any)**	300	636	237	399	301	63	37.3	82.7	-	-	-	-	-	-	-
**Cough (>=2 weeks)**	300	227	87	140	560	213	38.3	72.4	-	-	-	-	-	-	-
**Hb (<10 g/dL)**	300	644	231	413	287	69	35.9	80.6	-	-	-	-	-	-	-
**Hb (<8 g/dL)**	300	396	165	231	469	135	41.7	77.6	-	-	-	-	-	-	-
**BMI (<18.5 kg/m²)**	300	437	171	266	434	129	39.1	77.1	-	-	-	-	-	-	-
**Lymphadenopathy**	300	127	36	91	609	264	28.3	69.8	-	-	-	-	-	-	-
**W4SS or CRP (>=10 mg/L)¶**	300	965	300	665	35	0	31.1	100	-	-	-	-	-	-	-
**W4SS or CXR (abnormal)¶**	300	921	270	651	49	30	29.3	62	-	-	-	-	-	-	-
**W4SS then CRP (>=5 mg/L)¶**	300	845	285	560	140	15	33.7	90.3	-	-	-	-	-	-	-
**WHO Xpert algorithm*†**	300	-	-	-	-	-	-	-	228	651	49	72	82.3	90	4

		Outcome of screening§§							Outcome of screening then diagnosis§§						
Test	Total TB	TP+FP‡	TP	FP	TN	FN	PPV	NPV	TP	TN	FP	FN	PPV	NPV	NNS
**Xpert alone*†**	300	-	-	-	-	-	-	-	234	651	49	66	82.7	90.8	4

§According to WHO screening & diagnostic algorithm, Xpert testing is advised if an inpatient has a positive W4SS (defined as one or more of the following: current cough, fever, night sweats, or weight loss)

§§Estimated using the pooled point estimates for sensitivity and specificity for different tests/strategies

‡TP+FP is the number of participants who screen positive (i.e., the number who need subsequent diagnostic testing)

¶For parallel strategies, two screening tests are offered at the same time. For sequential strategies, a second screening test is offered only if the first screening test is positive

*Accuracy measures for entire algorithm using sputum and/or urine Xpert result

†The test accuracy of Xpert in those who were W4SS positive was: 4 studies; 586 participants; sensitivity 0.78 (0.68-0.86), specificity 0.93 (0.87-0.96).

Definition of abbreviations: BMI = body mass index, CRP = C-reactive protein, CXR = chest X-ray, FN= false negative, FP = false positive, Hb = haemoglobin, NNS = number needed to screen, NPV = negative predictive value, PPV = positive predictive value, TB = tuberculosis, TN = true negative, TP = true positive, W4SS = WHO four-symptom screen

Table S9B - Translation of pooled sensitivity and specificity estimates of different screening tests/strategies to a population of 1000 persons using Xpert as a reference standard§

		Outcome of screening§§						
**Test**	**Total TB**	**TP+FP‡**	**TP**	**FP**	**TN**	**FN**	**PPV**	**NPV**
	5% prevalence							
**W4SS**	50	904	49	855	95	1	5.4	99
**CRP (>=10 mg/L)**	50	807	47	760	190	3	5.8	98.4
**CRP (>=8 mg/L)**	50	836	47	788	162	3	5.6	98.2
**CRP (>=5 mg/L)**	50	884	48	836	114	2	5.4	98.3
**CXR (abnormal)**	50	604	34	570	380	16	5.7	96.1
**Cough (any)**	50	555	42	513	437	8	7.6	98.2
**Cough (>=2 weeks)**	50	201	21	180	770	29	10.4	96.4
**Hb (<10 g/dL)**	50	530	36	494	456	14	6.7	96.9
**Hb (<8 g/dL)**	50	271	24	247	703	26	8.9	96.4
**BMI (<18.5 kg/m²)**	50	396	25	370	580	25	6.3	95.9
**Lymphadenopathy**	50	107	12	95	855	38	11.2	95.7
**W4SS or CRP (>=10 mg/L)¶**	50	952	50	902	48	0	5.2	100
**W4SS or CXR (abnormal)¶**	50	958	46	912	38	4	4.9	91.6
**W4SS then CRP (>=5 mg/L)¶**	50	807	47	760	190	3	5.8	98.4
	10% prevalence							
**W4SS**	100	908	98	810	90	2	10.8	97.8
**CRP (>=10 mg/L)**	100	814	94	720	180	6	11.5	96.8
**CRP (>=8 mg/L)**	100	841	94	747	153	6	11.2	96.2
**CRP (>=5 mg/L)**	100	888	96	792	108	4	10.8	96.4
**CXR (abnormal)**	100	609	69	540	360	31	11.3	92.1
**Cough (any)**	100	570	84	486	414	16	14.7	96.3
**Cough (>=2 weeks)**	100	213	42	171	729	58	19.7	92.6
**Hb (<10 g/dL)**	100	539	71	468	432	29	13.2	93.7
**Hb (<8 g/dL)**	100	282	48	234	666	52	17	92.8
**BMI (<18.5 kg/m²)**	100	401	50	351	549	50	12.5	91.7
**Lymphadenopathy**	100	114	24	90	810	76	21.1	91.4
**W4SS or CRP (>=10 mg/L)¶**	100	955	100	855	45	0	10.5	100
**W4SS or CXR (abnormal)¶**	100	957	93	864	36	7	9.7	83.7
**W4SS then CRP (>=5 mg/L)¶**	100	814	94	720	180	6	11.5	96.8
	20% prevalence							
**W4SS**	200	916	196	720	80	4	21.4	95.2
**CRP (>=10 mg/L)**	200	828	188	640	160	12	22.7	93
**CRP (>=8 mg/L)**	200	852	188	664	136	12	22.1	91.9
**CRP (>=5 mg/L)**	200	896	192	704	96	8	21.4	92.3

		Outcome of screening§§						
**Test**	**Total TB**	**TP+FP‡**	**TP**	**FP**	**TN**	**FN**	**PPV**	**NPV**
**CXR (abnormal)**	200	618	138	480	320	62	22.3	83.8
**Cough (any)**	200	600	168	432	368	32	28	92
**Cough (>=2 weeks)**	200	236	84	152	648	116	35.6	84.8
**Hb (<10 g/dL)**	200	558	142	416	384	58	25.4	86.9
**Hb (<8 g/dL)**	200	304	96	208	592	104	31.6	85.1
**BMI (<18.5 kg/m²)**	200	412	100	312	488	100	24.3	83
**Lymphadenopathy**	200	128	48	80	720	152	37.5	82.6
**W4SS or CRP (>=10 mg/L)¶**	200	960	200	760	40	0	20.8	100
**W4SS or CXR (abnormal)¶**	200	954	186	768	32	14	19.5	69.6
**W4SS then CRP (>=5 mg/L)¶**	200	828	188	640	160	12	22.7	93
	30% prevalence							
**W4SS**	300	924	294	630	70	6	31.8	92.1
**CRP (>=10 mg/L)**	300	842	282	560	140	18	33.5	88.6
**CRP (>=8 mg/L)**	300	863	282	581	119	18	32.7	86.9
**CRP (>=5 mg/L)**	300	904	288	616	84	12	31.9	87.5
**CXR (abnormal)**	300	627	207	420	280	93	33	75.1
**Cough (any)**	300	630	252	378	322	48	40	87
**Cough (>=2 weeks)**	300	259	126	133	567	174	48.6	76.5
**Hb (<10 g/dL)**	300	577	213	364	336	87	36.9	79.4
**Hb (<8 g/dL)**	300	326	144	182	518	156	44.2	76.9
**BMI (<18.5 kg/m²)**	300	423	150	273	427	150	35.5	74
**Lymphadenopathy**	300	142	72	70	630	228	50.7	73.4
**W4SS or CRP (>=10 mg/L)¶**	300	965	300	665	35	0	31.1	100
**W4SS or CXR (abnormal)¶**	300	951	279	672	28	21	29.3	57.1
**W4SS then CRP (>=5 mg/L)¶**	300	842	282	560	140	18	33.5	88.6

§§Estimated using the pooled point estimates for sensitivity and specificity for different tests/strategies

‡TP+FP is the number of participants who screen positive (i.e., the number who need subsequent diagnostic testing)

¶For parallel strategies, two screening tests are offered at the same time. For sequential strategies, a second screening test is offered only if the first screening test is positive

Definition of abbreviations: BMI = body mass index, CRP = C-reactive protein, CXR = chest X-ray, FN= false negative, FP = false positive, Hb = haemoglobin, NNS = number needed to screen, NPV = negative predictive value, PPV = positive predictive value, TB = tuberculosis, TN = true negative, TP = true positive, W4SS = WHO four-symptom screen

Table S10 - Sensitivity analyses of diagnostic accuracy (pooled sensitivity and specificity) for each screening test/strategy for the detection of tuberculosis using an alternative reference standard of culture or Xpert*

	Sensitivity analyses 1*				Sensitivity analyses 2**			
	No of studies	N	Sensitivity (95% CI)	Specificity (95% CI)	No of studies	N	Sensitivity (95% CI)	Specificity (95% CI)
**W4SS**	6	2,211	99 (93-100)	9 (6-13)	4	697	97 (91-99)	8 (4-17)
**CRP (>=10 mg/L)**	1	400	94 (88-97)	22 (17-27)	1	400	94 (88-97)	22 (17-27)
**CRP (>=8 mg/L)**	1	400	94 (88-97)	19 (14-24)	1	400	94 (88-97)	19 (14-24)
**CRP (>=5 mg/L)**	1	400	96 (91-98)	13 (9-17)	1	400	96 (91-98)	13 (9-17)
**CXR (abnormal)**	2	176	69 (43-86)	41 (33-48)	1	52	57 (23-86)	42 (29-57)
**Cough (any)**	6	2,208	83 (69-92)	46 (37-55)	4	694	75 (59-86)	45 (33-58)
**Cough (>=2 weeks)**	4	1,895	41 (20-65)	81 (62-92)	3	631	30 (14-53)	81 (53-94)
**Hb (<10 g/dL)**	5	2,037	73 (66-80)	47 (37-58)	3	527	73 (66-80)	41 (29-56)
**Hb (<8 g/dL)**	5	2,037	53 (42-64)	72 (63-80)	3	527	56 (34-77)	68 (54-80)
**BMI (<18.5 kg/m^2)**	4	1,571	50 (41-59)	61 (49-71)	2	112	56 (33-76)	63 (53-72)
**Lymphadenopathy**	3	356	22 (13-35)	90 (86-93)	2	123	14 (4-35)	87 (79-92)
**W4SS or CRP (>=10 mg/L)¶**	1	399	100 (94-100)	5 (3-8)	1	399	100 (94-100)	5 (3-8)
**W4SS or CXR (abnormal)¶**	2	176	94 (66-99)	4 (2-9)	1	52	86 (42-98)	4 (1-16)
**W4SS then CRP (>=5 mg/L)¶**	1	399	93 (87-96)	21 (17-26)	1	399	93 (87-96)	21 (17-26)

*Reference standard of culture or Xpert of sputum and/or other specimens

*Reference standard of culture or Xpert of sputum and/or other specimens among datasets that collected sputum for culture

¶For parallel strategies, two screening tests are offered at the same time. For sequential strategies, a second screening test is offered only if the first screening test is positive

Definition of abbreviations: BMI = body mass index, CRP = C-reactive protein, CXR = chest X-ray, Hb = haemoglobin, W4SS = WHO four-symptom screen

Table S11 - Diagnostic yield of different Xpert tests and sample types as a proportion of total microbiologically confirmed tuberculosis cases†

Study	Bjerrum‡	Gupta-Wright intervention*	Gupta-Wright control*	Heidebrecht	Huerga	Lawn**	Thit
**Total sample size**	69	1287	1287	156	387	420	54
**Microbiological sample available**	69	1279	779	156	235	420	54
**Microbiologically confirmed¶**	15	122	82	41	33	139	7
**Sputum culture + (%)**	13 (87%)	-	-	31 (76%)	-	58 (42%)	4 (57%)
**N**	69	-	-	131	-	209	54
**Non-sputum culture + (%)**	-	-	-	-	-	70 (50%)	-
**N**	-	-	-	-	-	420	-
**Total culture + (%)**	13 (87%)	-	-	31 (76%)	-	109 (78%)	4 (57%)
**N**	69	-	-	131	-	420	54
**Sputum Xpert + (%)**	9 (60%)	85 (70%)	82 (100%)	35 (85%)	33 (100%)	57 (41%)	4 (57%)
**N**	50	832	779	146	235	195	54
**Urine Xpert + (%)**	-	74 (61%)	-	-	-	89 (64%)	-
**N**	-	1270	-	-	-	411	-
**Total Xpert + (%)**	9 (60%)	122 (100%)	82 (100%)	35 (85%)	33 (100%)	116 (83%)	4 (57%)
**N**	50	1279	779	146	235	414	54

†Denominator for % is microbiologically confirmed

‡Study by Bjerrum et al has fewer samples collected for Xpert testing because Xpert only became available after study enrollment began

*Study by Gupta-Wright et al (2018) involved an intervention arm (systematically collected concentrated urine Xpert and sputum Xpert) and control arm (systematically collected sputum Xpert only)

**The number (%) of all microbiologically cases diagnosed with concentrated urine Xpert was 82 (59%; 402 participants) and with unconcentrated urine Xpert was 59 (42%; 405 participants).

¶Defined as any Xpert, or culture positive.

Figure S1 - Forest plots of sensitivity and specificity estimates for each screening test/strategy (C-reactive protein >=8 mg/L omitted)

Figure S1A - Forest plots of sensitivity and specificity estimates for each screening test/strategy using culture as a reference standard

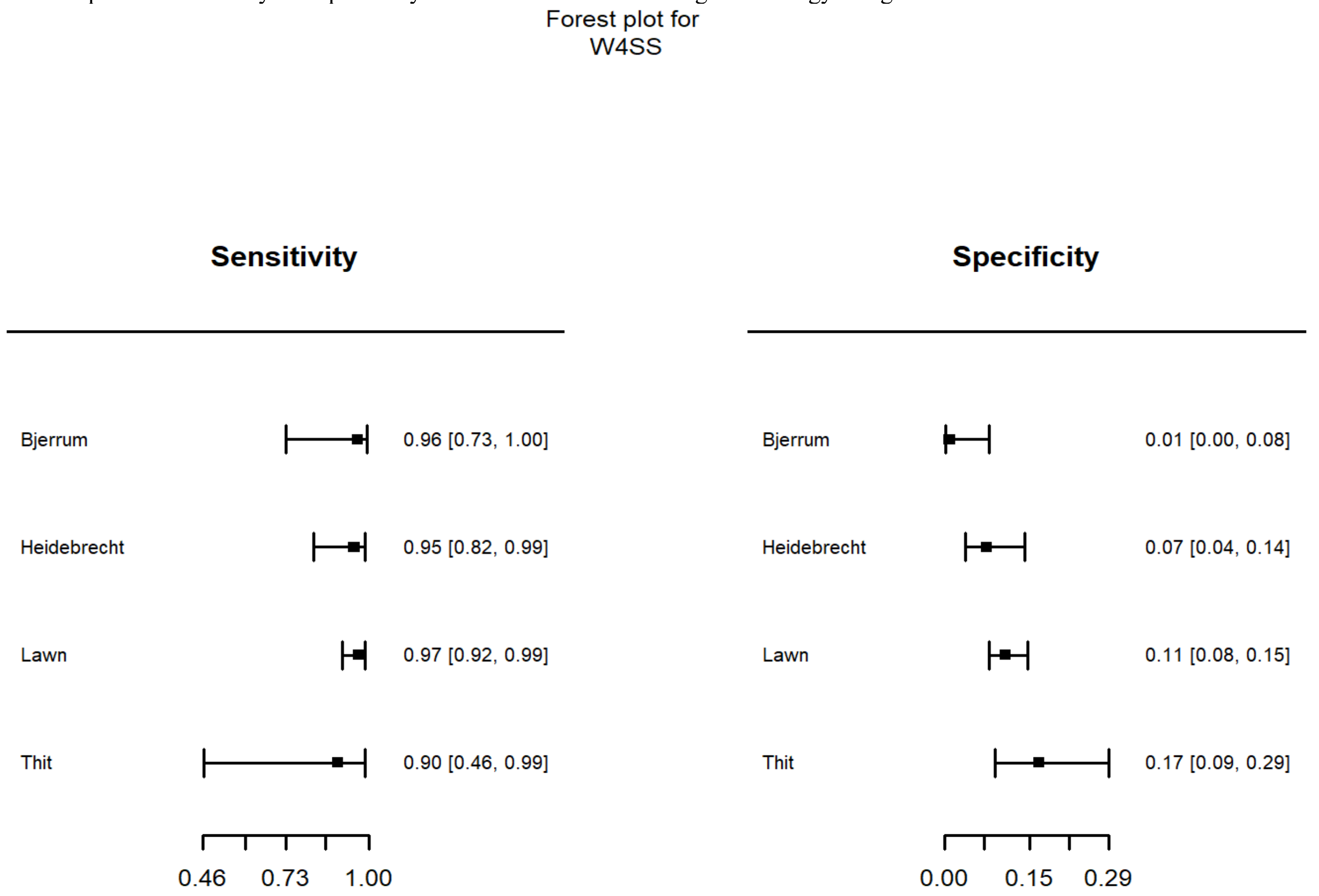

Forest plot for
Top: CRP (>=10 mg/L) and Bottom: CRP (>=5 mg/L)

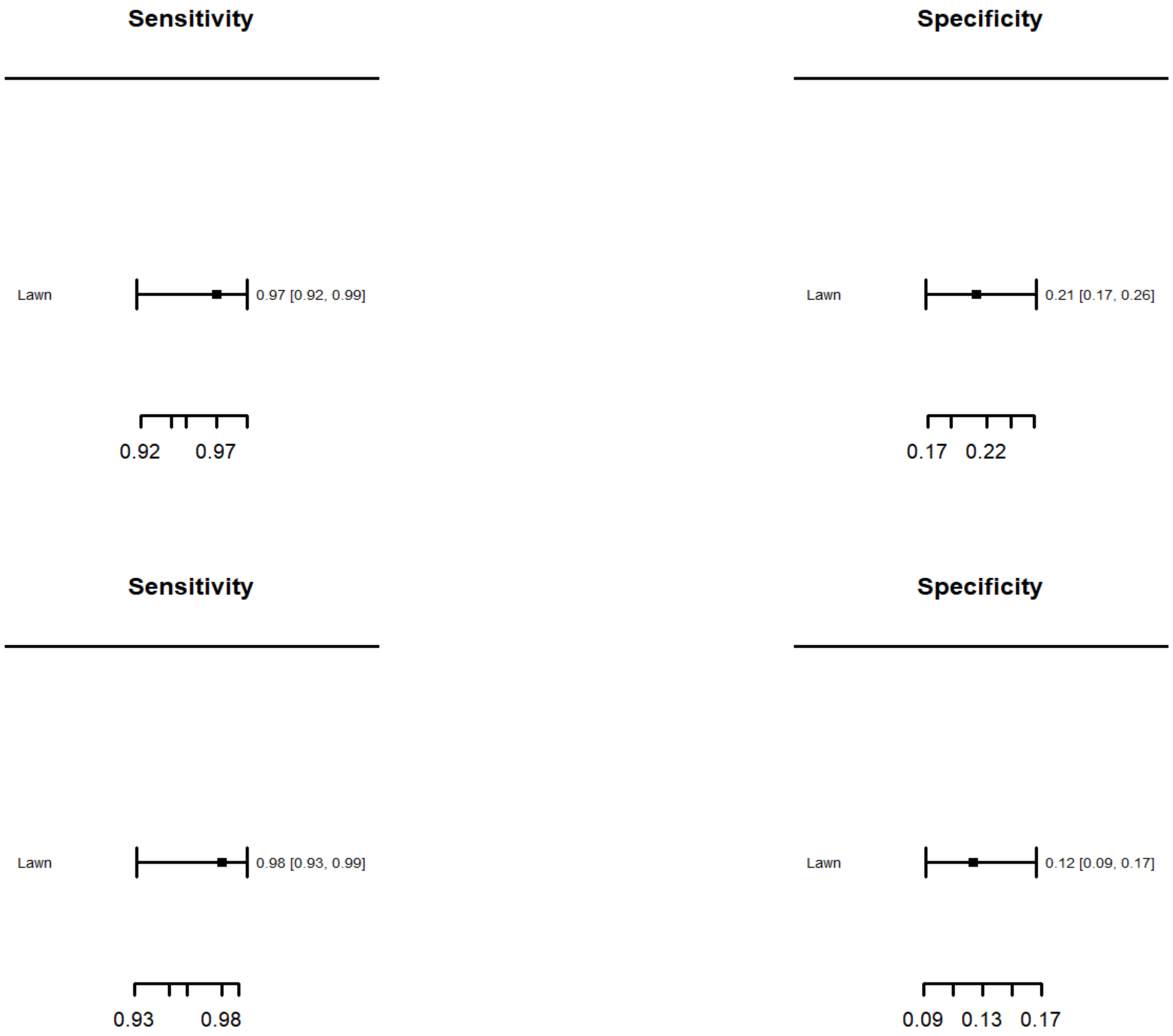

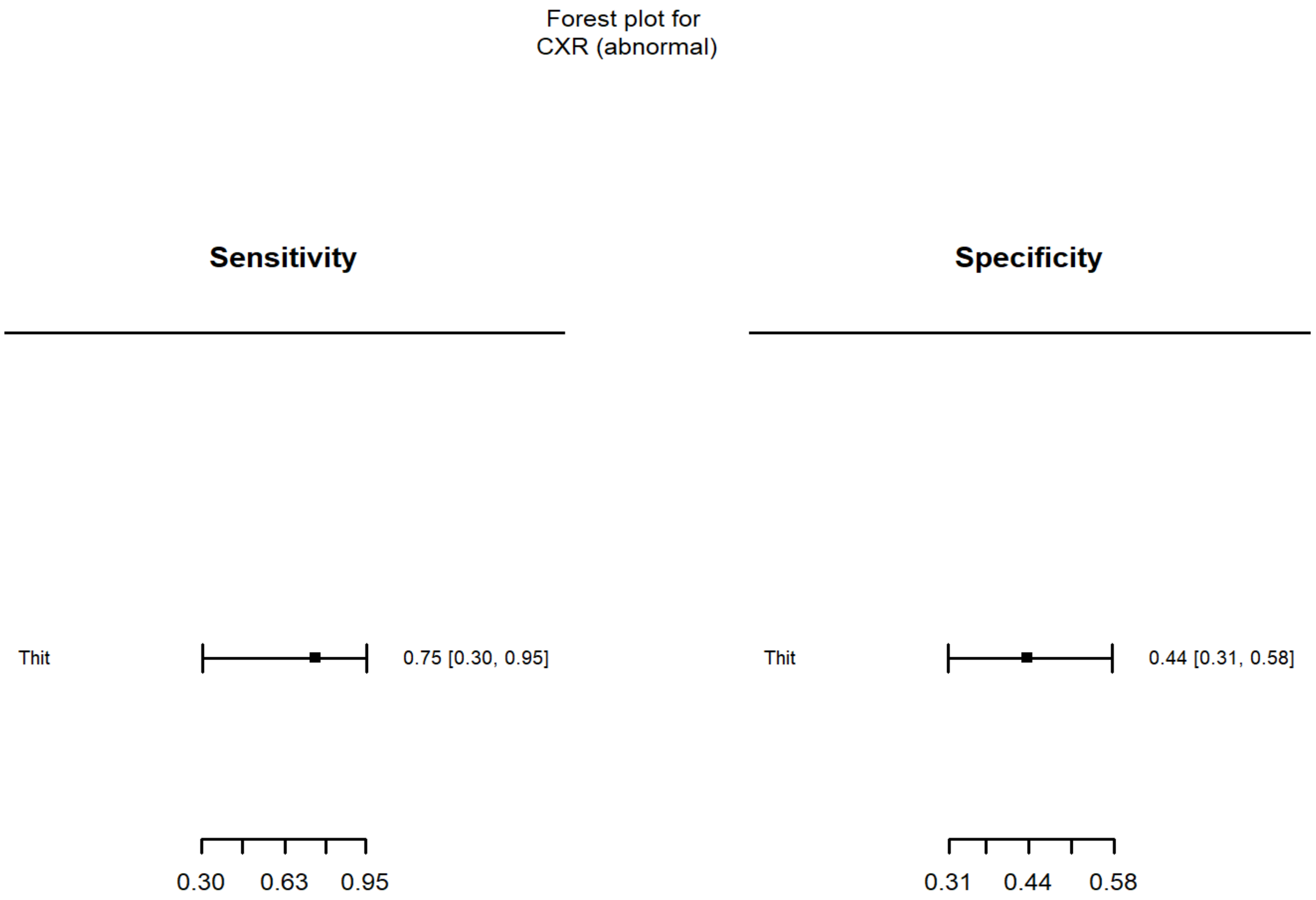
Forest plot for
CXR (abnormal)
Sensitivity
Specificity
Thit
0.75 [0.30, 0.95]
Thit
0.44 [0.31, 0.58]
0.30
0.63
0.95
0.31
0.44
0.58

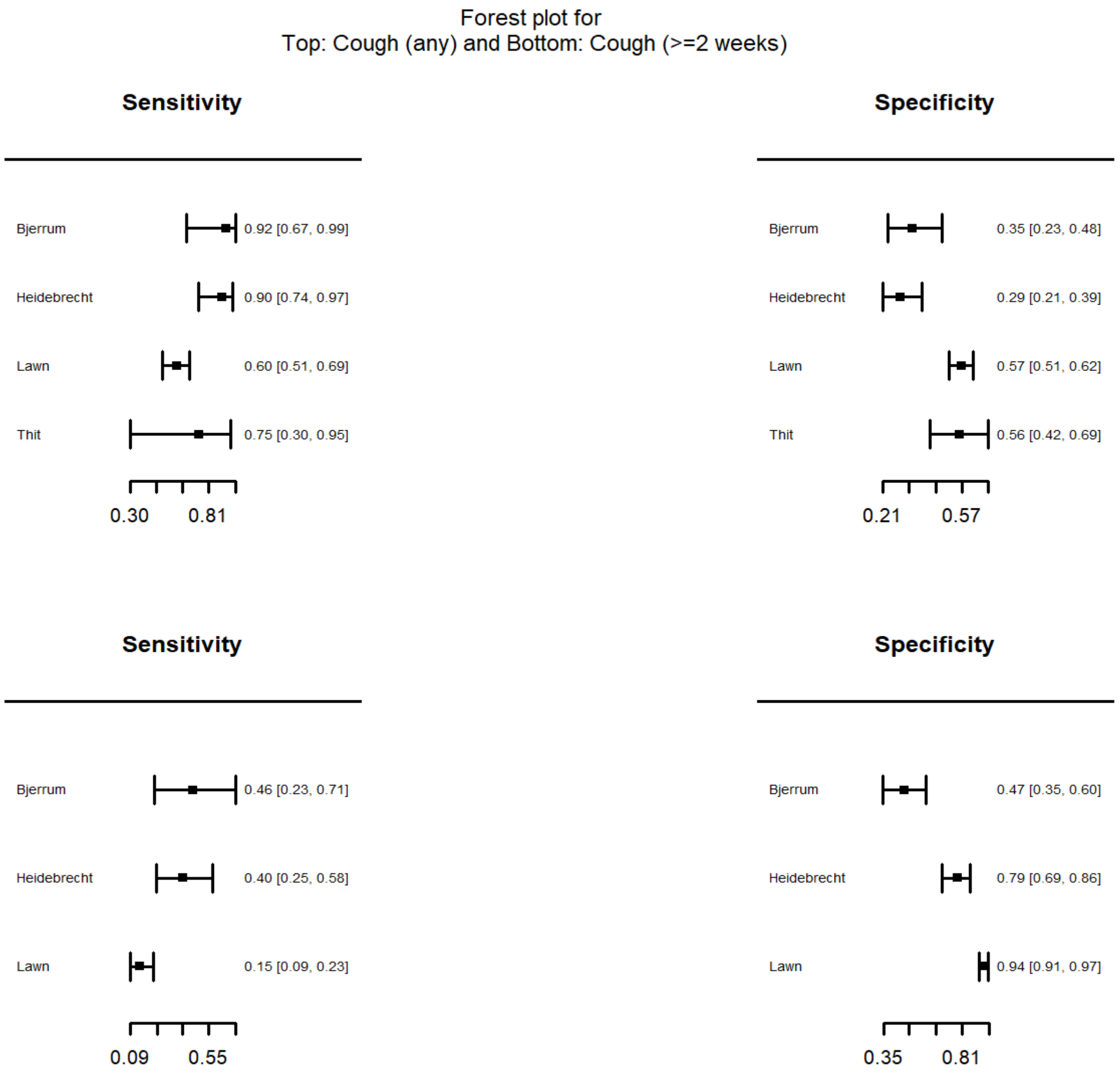

Forest plot for
Top: Cough (any) and Bottom: Cough (>=2 weeks)
Sensitivity
Bjerrum 0.92 [0.67, 0.99]
Heidebrecht 0.90 [0.74, 0.97]
Lawn 0.60 [0.51, 0.69]
Thit 0.75 [0.30, 0.95]
0.30 0.81
Specificity
Bjerrum 0.35 [0.23, 0.48]
Heidebrecht 0.29 [0.21, 0.39]
Lawn 0.57 [0.51, 0.62]
Thit 0.56 [0.42, 0.69]
0.21 0.57
Sensitivity
Bjerrum 0.46 [0.23, 0.71]
Heidebrecht 0.40 [0.25, 0.58]
Lawn 0.15 [0.09, 0.23]
0.09 0.55
Specificity
Bjerrum 0.47 [0.35, 0.60]
Heidebrecht 0.79 [0.69, 0.86]
Lawn 0.94 [0.91, 0.97]
0.35 0.81

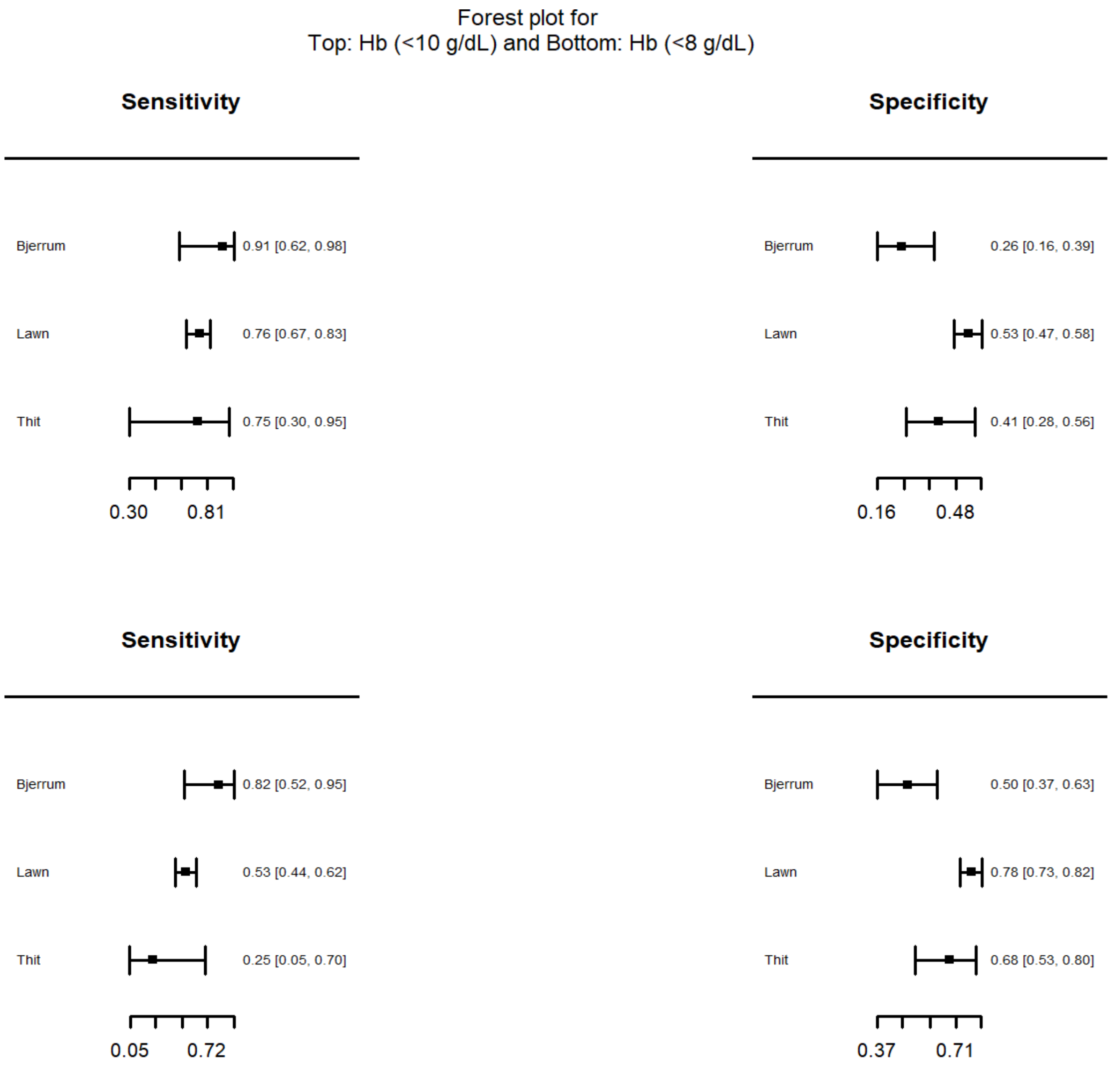
Forest plot for
Top: Hb (<10 g/dL) and Bottom: Hb (<8 g/dL)
Sensitivity
Specificity
Bjerrum
0.91 [0.62, 0.98]
0.26 [0.16, 0.39]
Lawn
0.76 [0.67, 0.83]
0.53 [0.47, 0.58]
Thit
0.75 [0.30, 0.95]
0.41 [0.28, 0.56]
0.30
0.81
0.16
0.48
Sensitivity
Specificity
Bjerrum
0.82 [0.52, 0.95]
0.50 [0.37, 0.63]
Lawn
0.53 [0.44, 0.62]
0.78 [0.73, 0.82]
Thit
0.25 [0.05, 0.70]
0.68 [0.53, 0.80]
0.05
0.72
0.37
0.71

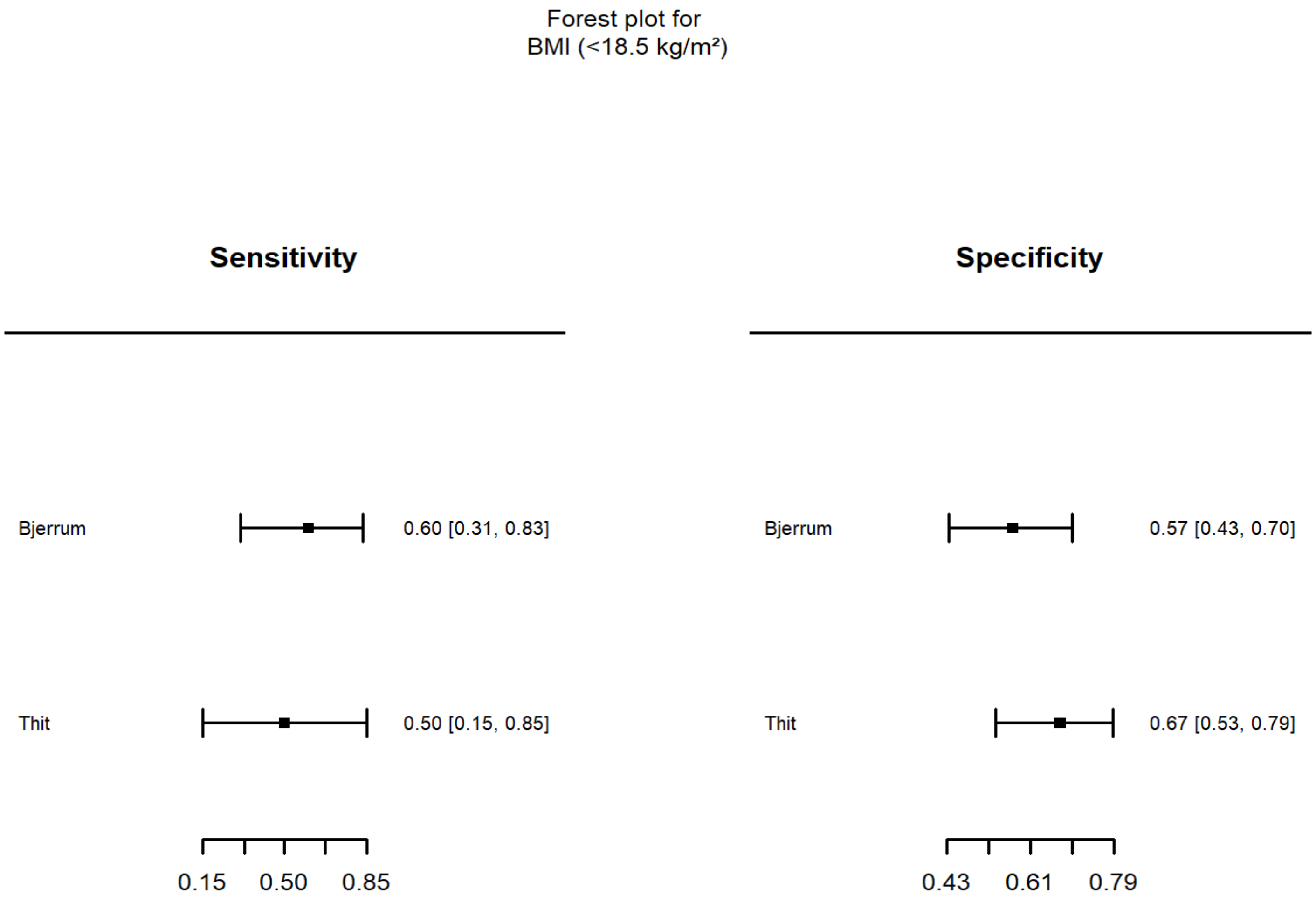
Forest plot for
BMI (<18.5 kg/m^2)
Sensitivity
Specificity
Bjerrum
0.60 [0.31, 0.83]
Bjerrum
0.57 [0.43, 0.70]
Thit
0.50 [0.15, 0.85]
Thit
0.67 [0.53, 0.79]
0.15 0.50 0.85
0.43 0.61 0.79

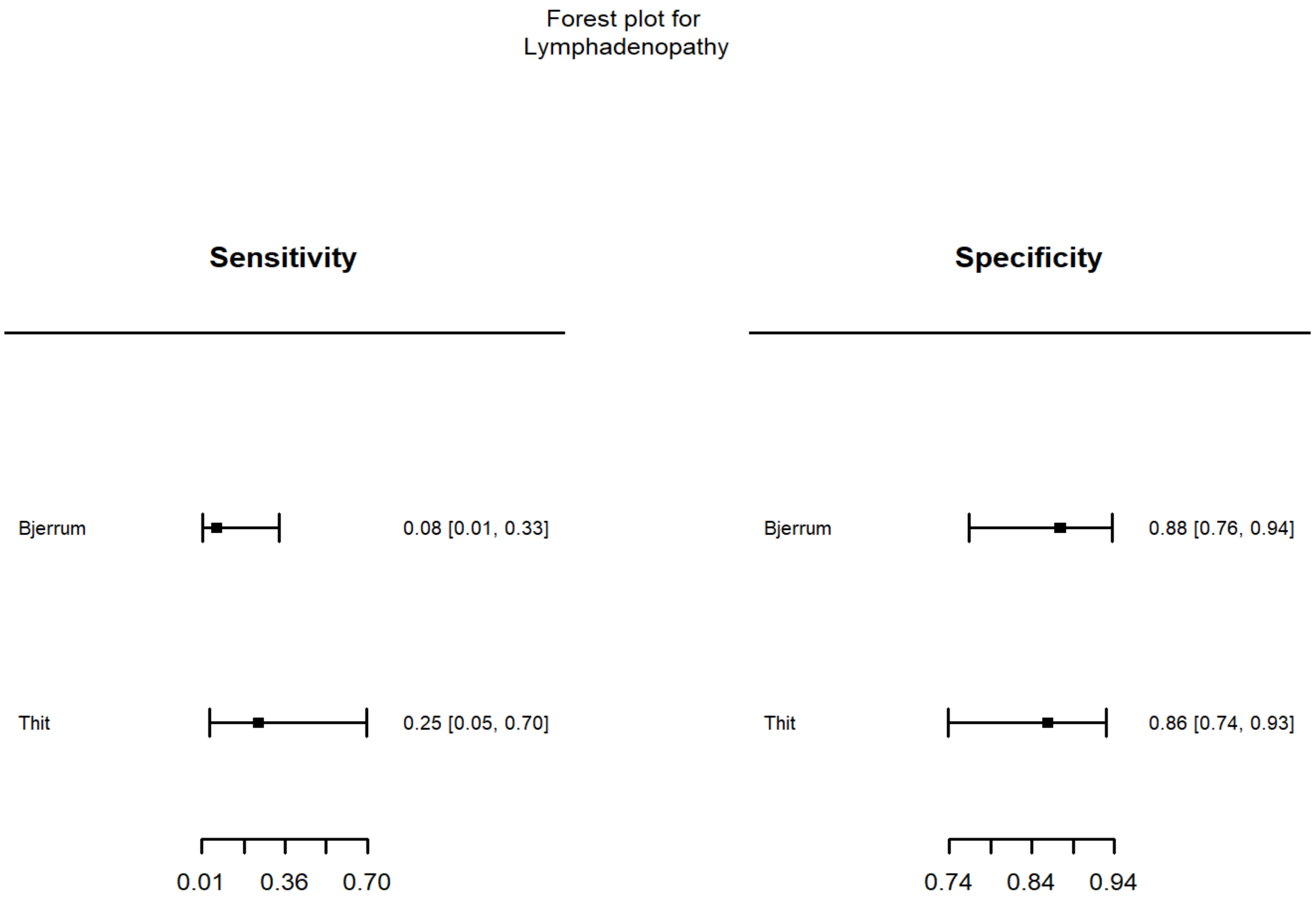
Forest plot for
Lymphadenopathy
Sensitivity
Specificity
Bjerrum
0.08 [0.01, 0.33]
Thit
0.25 [0.05, 0.70]
0.01
0.36
0.70
Bjerrum
0.88 [0.76, 0.94]
Thit
0.86 [0.74, 0.93]
0.74
0.84
0.94

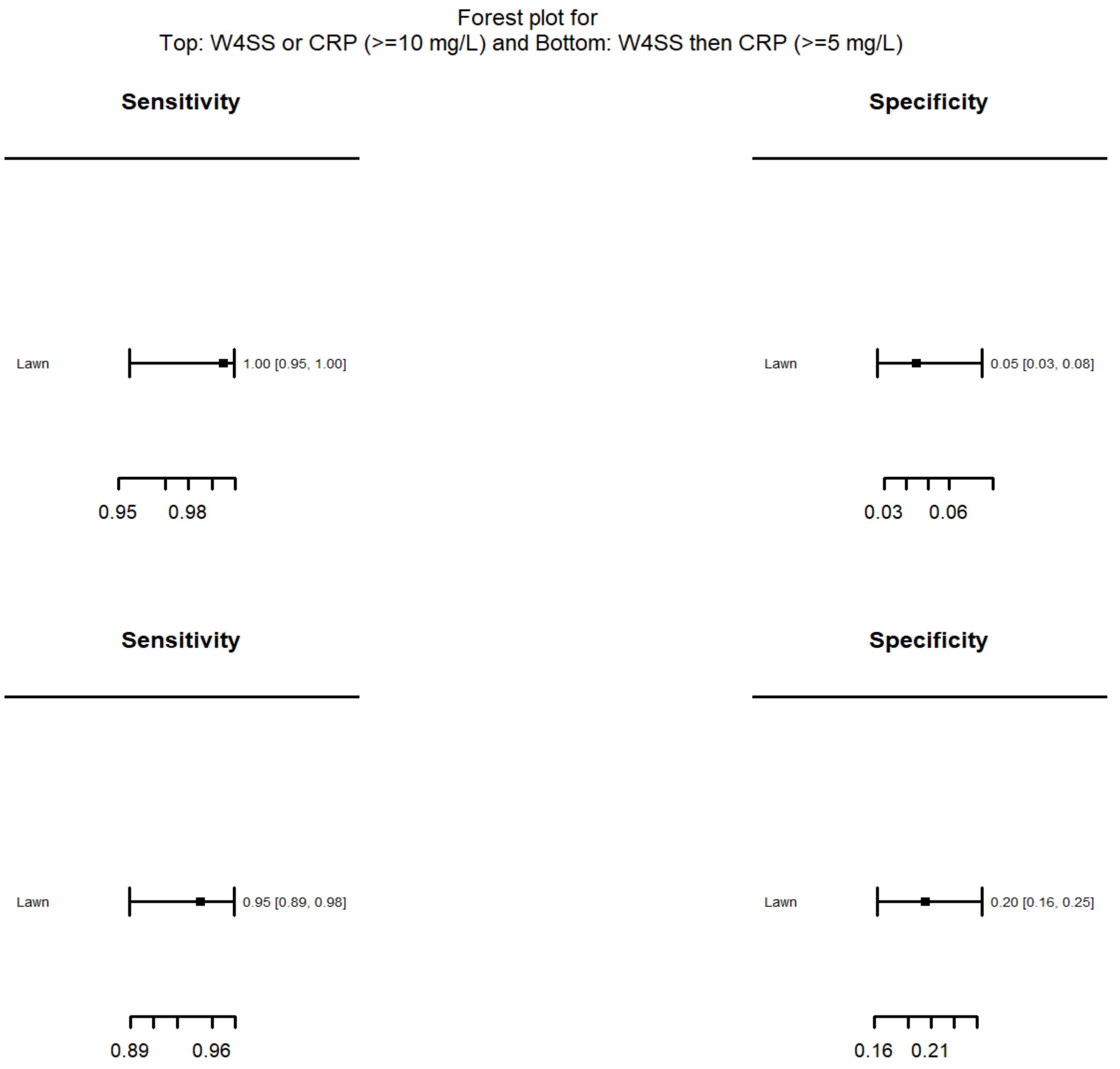
Forest plot for
Top: W4SS or CRP (>=10 mg/L) and Bottom: W4SS then CRP (>=5 mg/L)
Sensitivity
Specificity
Lawn
1.00 [0.95, 1.00]
Lawn
0.05 [0.03, 0.08]
0.95
0.98
0.03
0.06
Sensitivity
Specificity
Lawn
0.95 [0.89, 0.98]
Lawn
0.20 [0.16, 0.25]
0.89
0.96
0.16
0.21

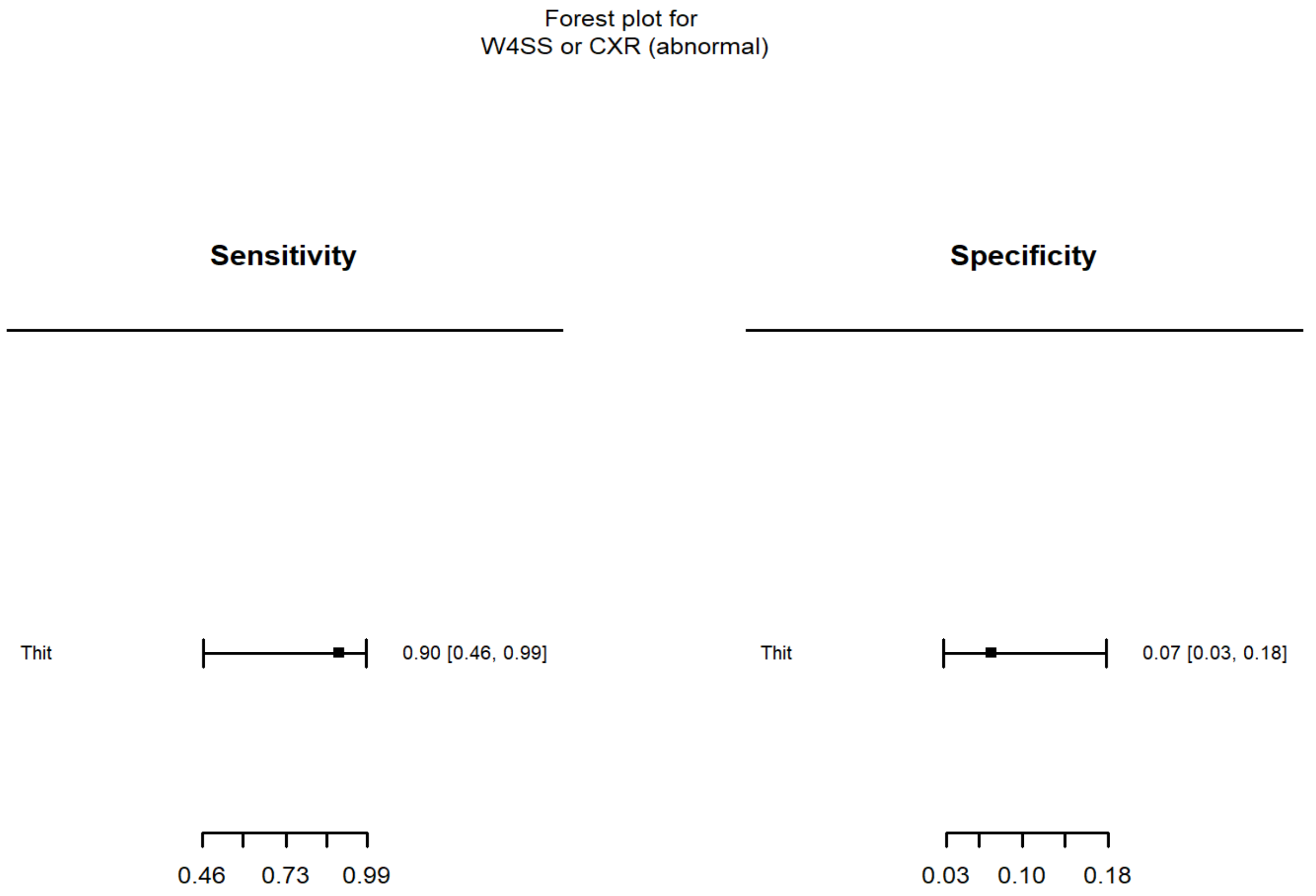
Forest plot for
W4SS or CXR (abnormal)
Sensitivity
Specificity
Thit
0.90 [0.46, 0.99]
Thit
0.07 [0.03, 0.18]
0.46
0.73
0.99
0.03
0.10
0.18

Forest plot for
Top: WHO Xpert algorithm and Bottom: Xpert alone

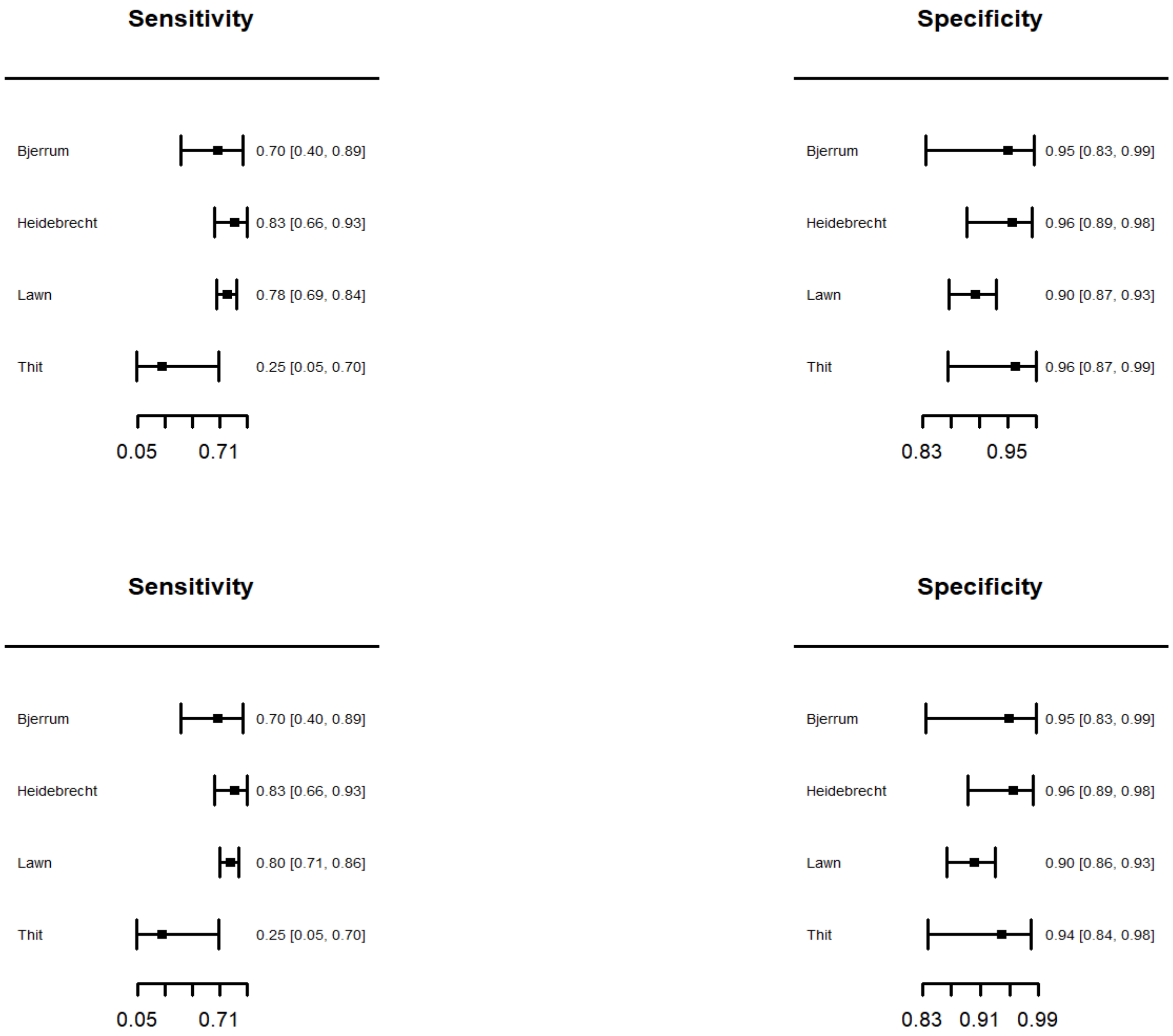

Figure S1B - Forest plots of sensitivity and specificity estimates for each screening test/strategy using Xpert as a reference standard

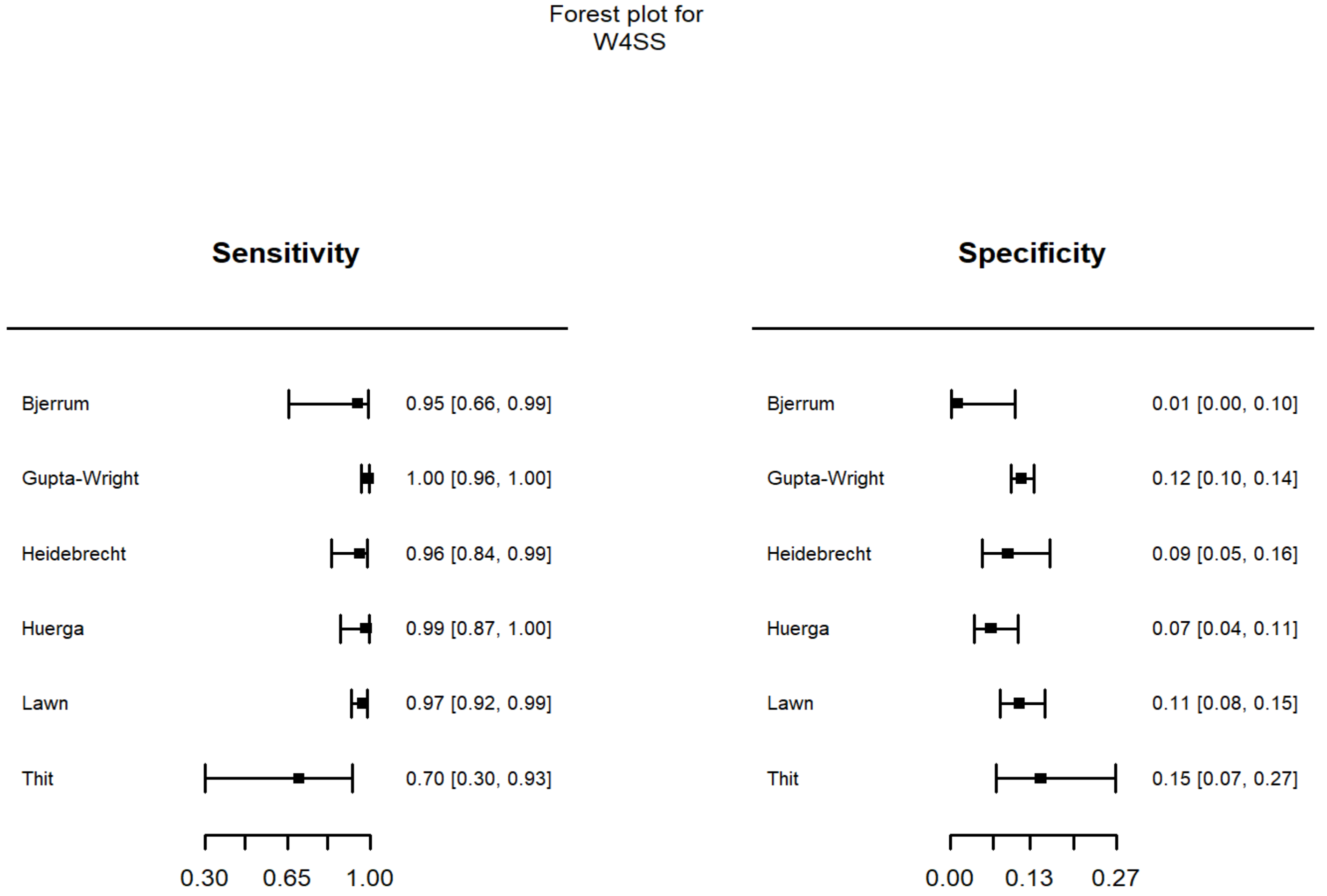

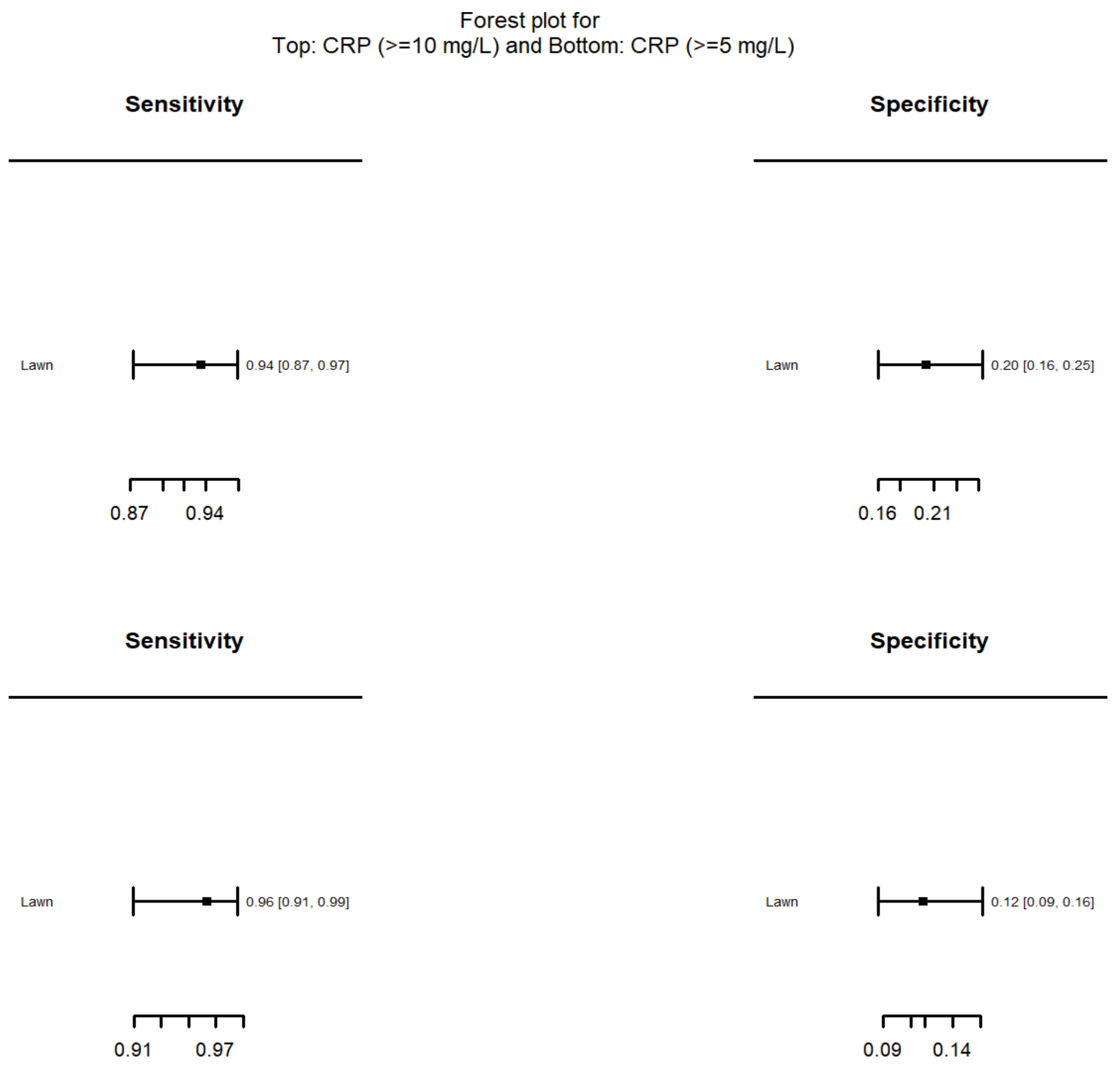
Forest plot for
Top: CRP (>=10 mg/L) and Bottom: CRP (>=5 mg/L)
Sensitivity
Specificity
Lawn
0.94 [0.87, 0.97]
0.87
0.94
Lawn
0.20 [0.16, 0.25]
0.16
0.21
Sensitivity
Specificity
Lawn
0.96 [0.91, 0.99]
0.91
0.97
Lawn
0.12 [0.09, 0.16]
0.09
0.14

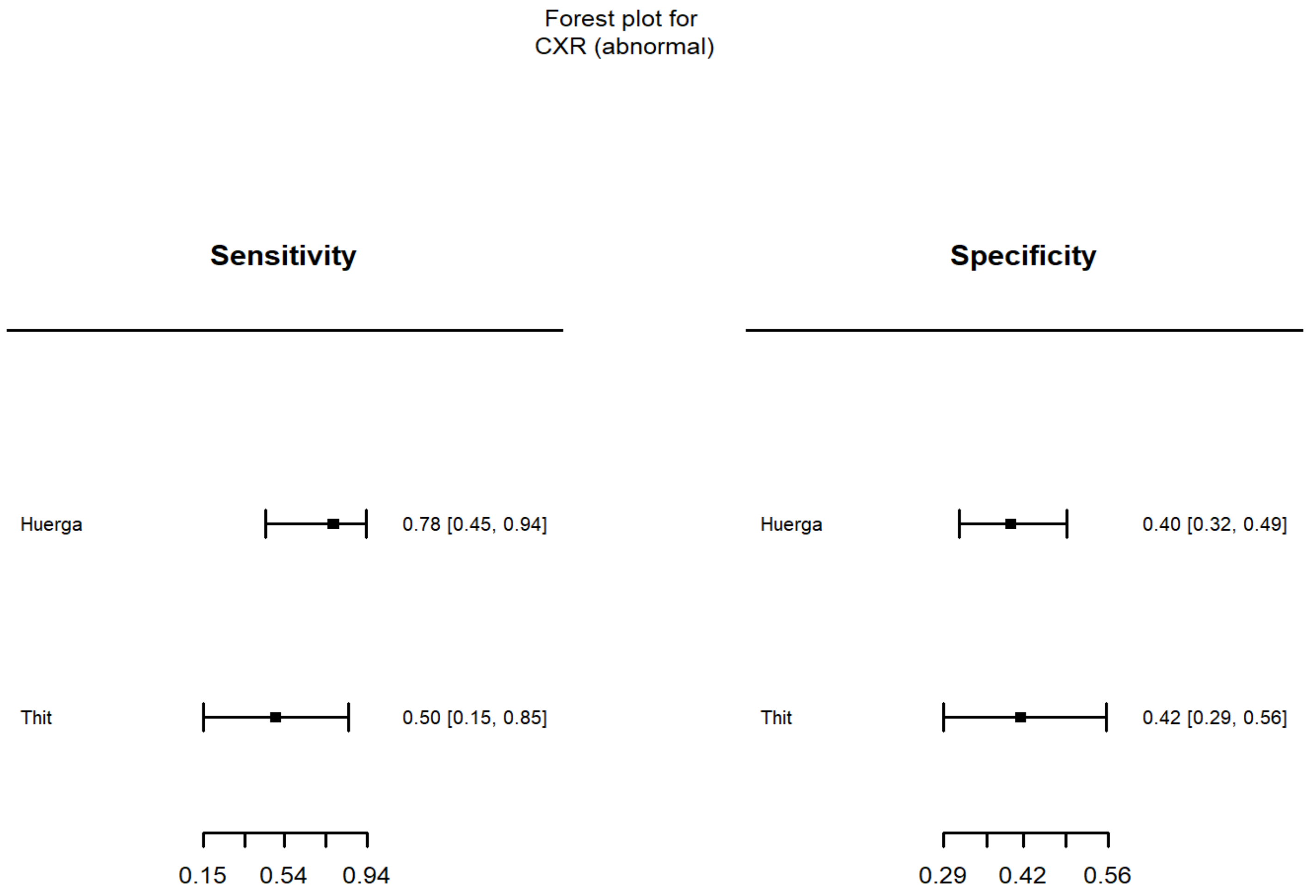
Forest plot for
CXR (abnormal)
Sensitivity
Specificity
Huerga
0.78 [0.45, 0.94]
0.40 [0.32, 0.49]
Thit
0.50 [0.15, 0.85]
0.42 [0.29, 0.56]
0.15
0.54
0.94
0.29
0.42
0.56

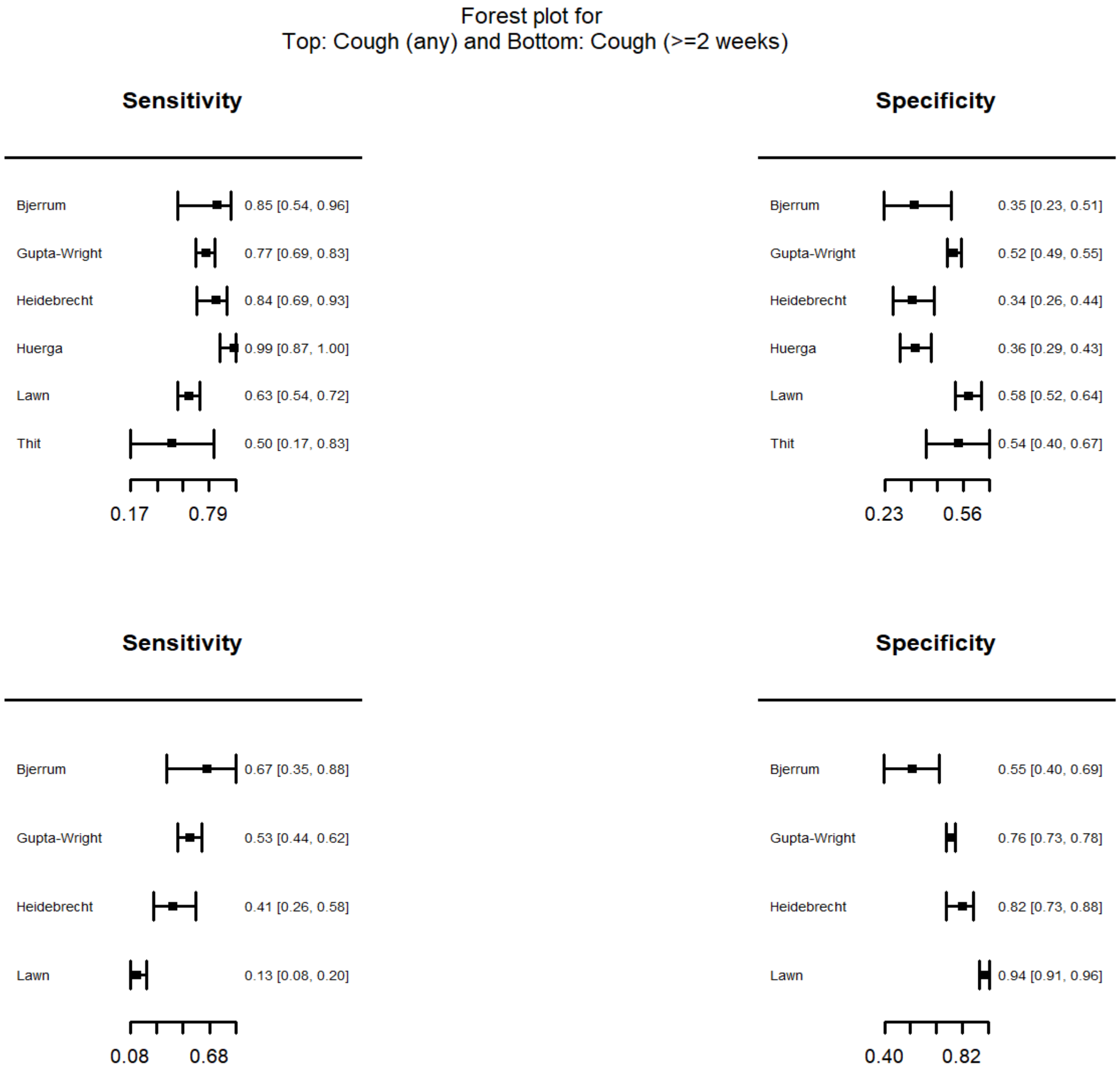
Forest plot for
Top: Cough (any) and Bottom: Cough (>=2 weeks)
Sensitivity
Bjerrum 0.85 [0.54, 0.96]
Gupta-Wright 0.77 [0.69, 0.83]
Heidebrecht 0.84 [0.69, 0.93]
Huerga 0.99 [0.87, 1.00]
Lawn 0.63 [0.54, 0.72]
Thit 0.50 [0.17, 0.83]
0.17 0.79
Specificity
Bjerrum 0.35 [0.23, 0.51]
Gupta-Wright 0.52 [0.49, 0.55]
Heidebrecht 0.34 [0.26, 0.44]
Huerga 0.36 [0.29, 0.43]
Lawn 0.58 [0.52, 0.64]
Thit 0.54 [0.40, 0.67]
0.23 0.56
Sensitivity
Bjerrum 0.67 [0.35, 0.88]
Gupta-Wright 0.53 [0.44, 0.62]
Heidebrecht 0.41 [0.26, 0.58]
Lawn 0.13 [0.08, 0.20]
0.08 0.68
Specificity
Bjerrum 0.55 [0.40, 0.69]
Gupta-Wright 0.76 [0.73, 0.78]
Heidebrecht 0.82 [0.73, 0.88]
Lawn 0.94 [0.91, 0.96]
0.40 0.82

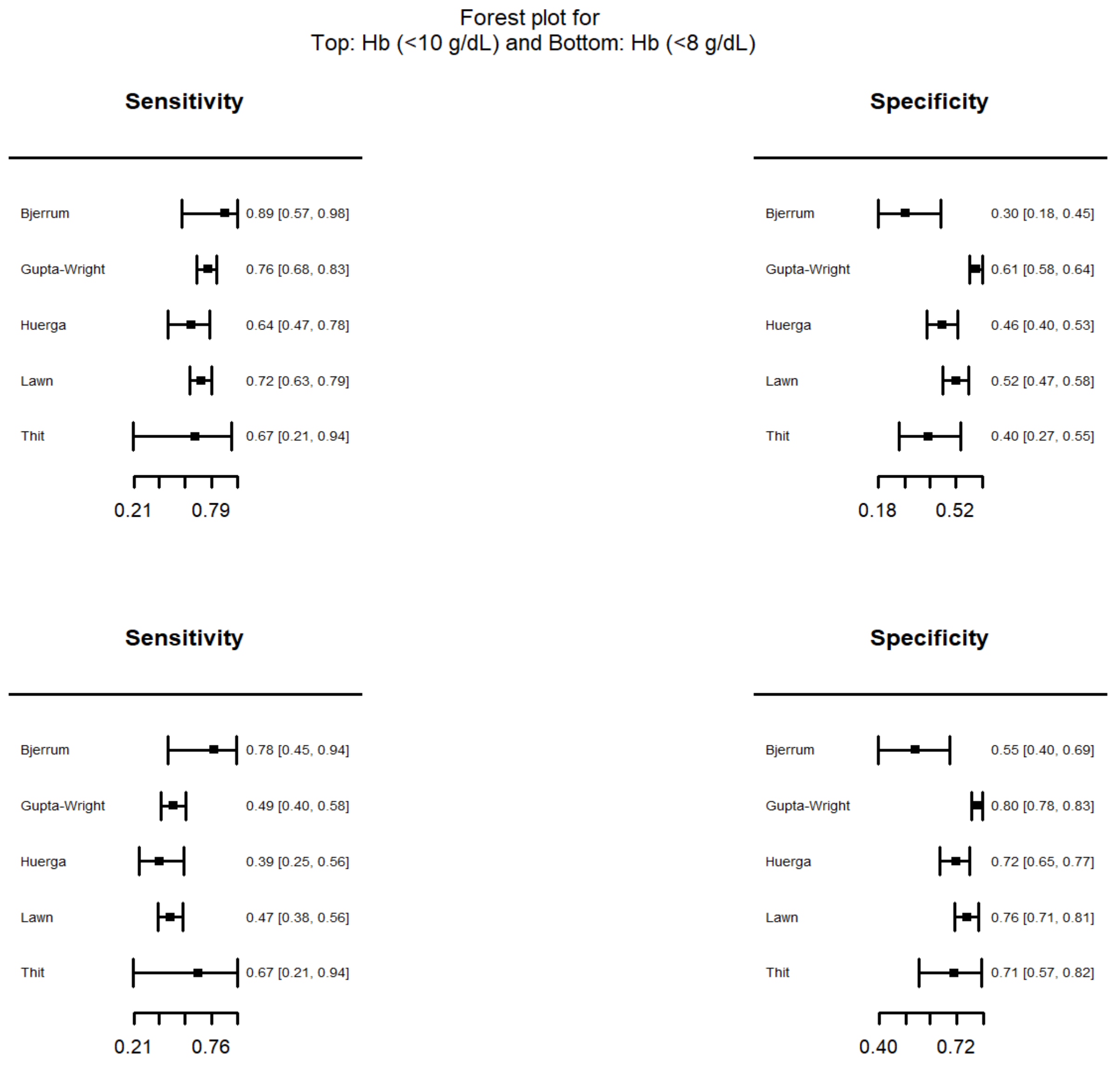
Forest plot for
Top: Hb (<10 g/dL) and Bottom: Hb (<8 g/dL)
Sensitivity
Bjerrum 0.89 [0.57, 0.98]
Gupta-Wright 0.76 [0.68, 0.83]
Huerga 0.64 [0.47, 0.78]
Lawn 0.72 [0.63, 0.79]
Thit 0.67 [0.21, 0.94]
0.21 0.79
Specificity
Bjerrum 0.30 [0.18, 0.45]
Gupta-Wright 0.61 [0.58, 0.64]
Huerga 0.46 [0.40, 0.53]
Lawn 0.52 [0.47, 0.58]
Thit 0.40 [0.27, 0.55]
0.18 0.52
Sensitivity
Bjerrum 0.78 [0.45, 0.94]
Gupta-Wright 0.49 [0.40, 0.58]
Huerga 0.39 [0.25, 0.56]
Lawn 0.47 [0.38, 0.56]
Thit 0.67 [0.21, 0.94]
0.21 0.76
Specificity
Bjerrum 0.55 [0.40, 0.69]
Gupta-Wright 0.80 [0.78, 0.83]
Huerga 0.72 [0.65, 0.77]
Lawn 0.76 [0.71, 0.81]
Thit 0.71 [0.57, 0.82]
0.40 0.72

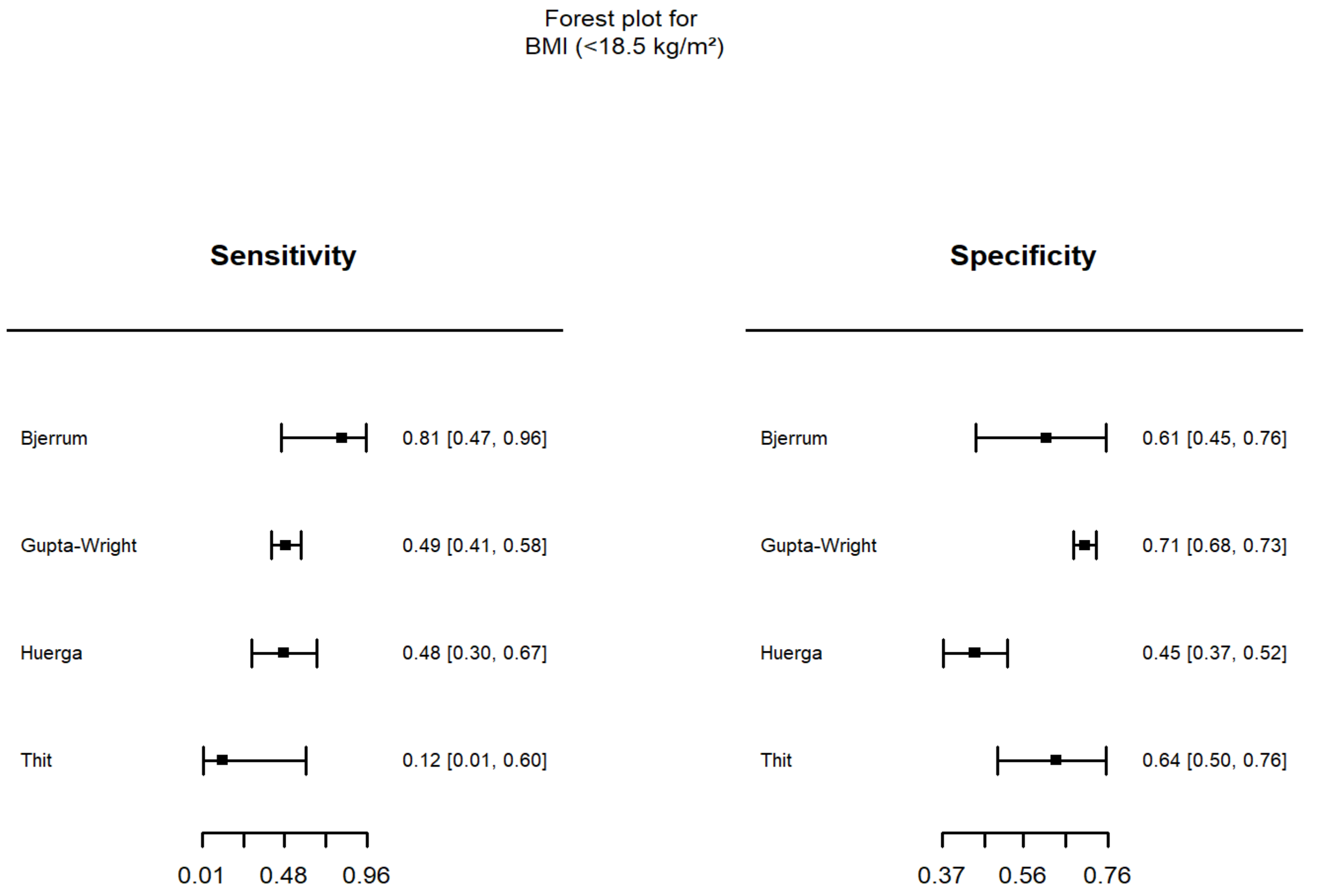
Forest plot for
BMI (<18.5 kg/m²)
Sensitivity
Specificity
Bjerrum
0.81 [0.47, 0.96]
Bjerrum
0.61 [0.45, 0.76]
Gupta-Wright
0.49 [0.41, 0.58]
Gupta-Wright
0.71 [0.68, 0.73]
Huerga
0.48 [0.30, 0.67]
Huerga
0.45 [0.37, 0.52]
Thit
0.12 [0.01, 0.60]
Thit
0.64 [0.50, 0.76]
0.01
0.48
0.96
0.37
0.56
0.76

Forest plot for
Lymphadenopathy

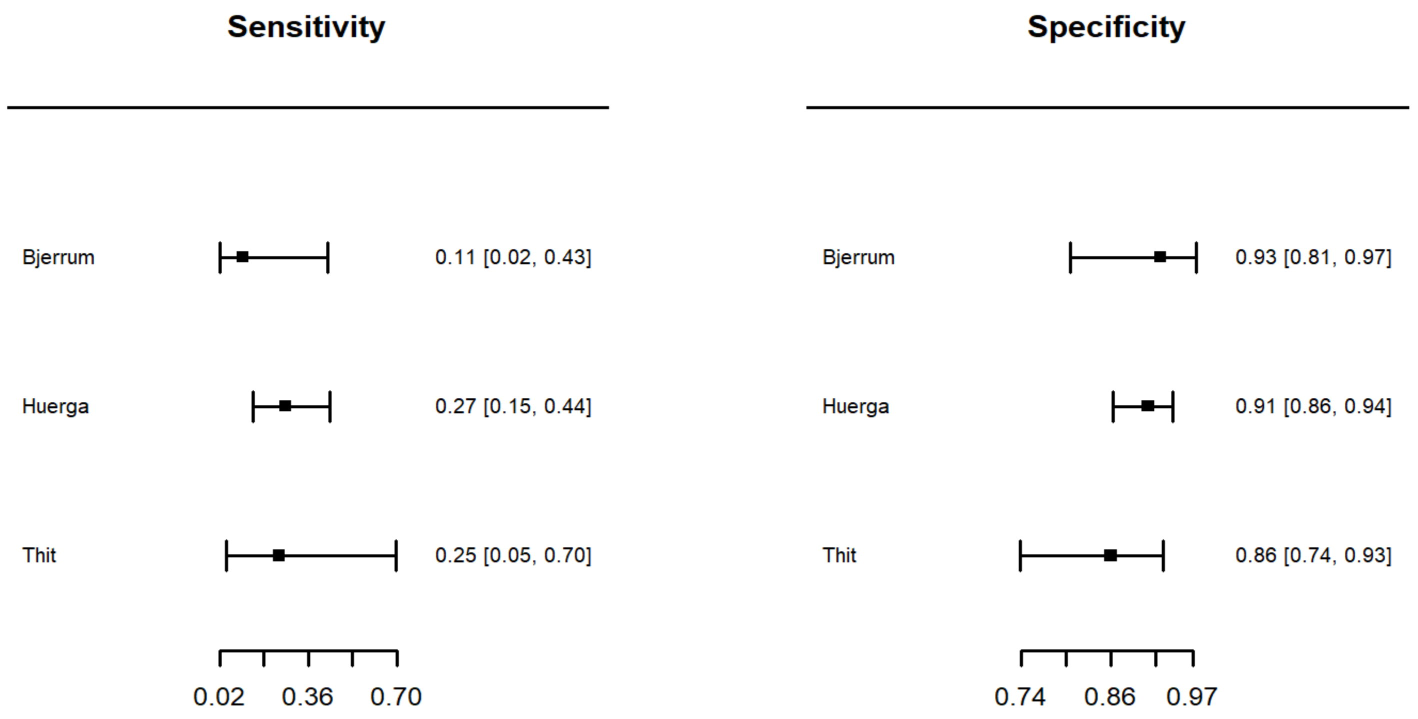

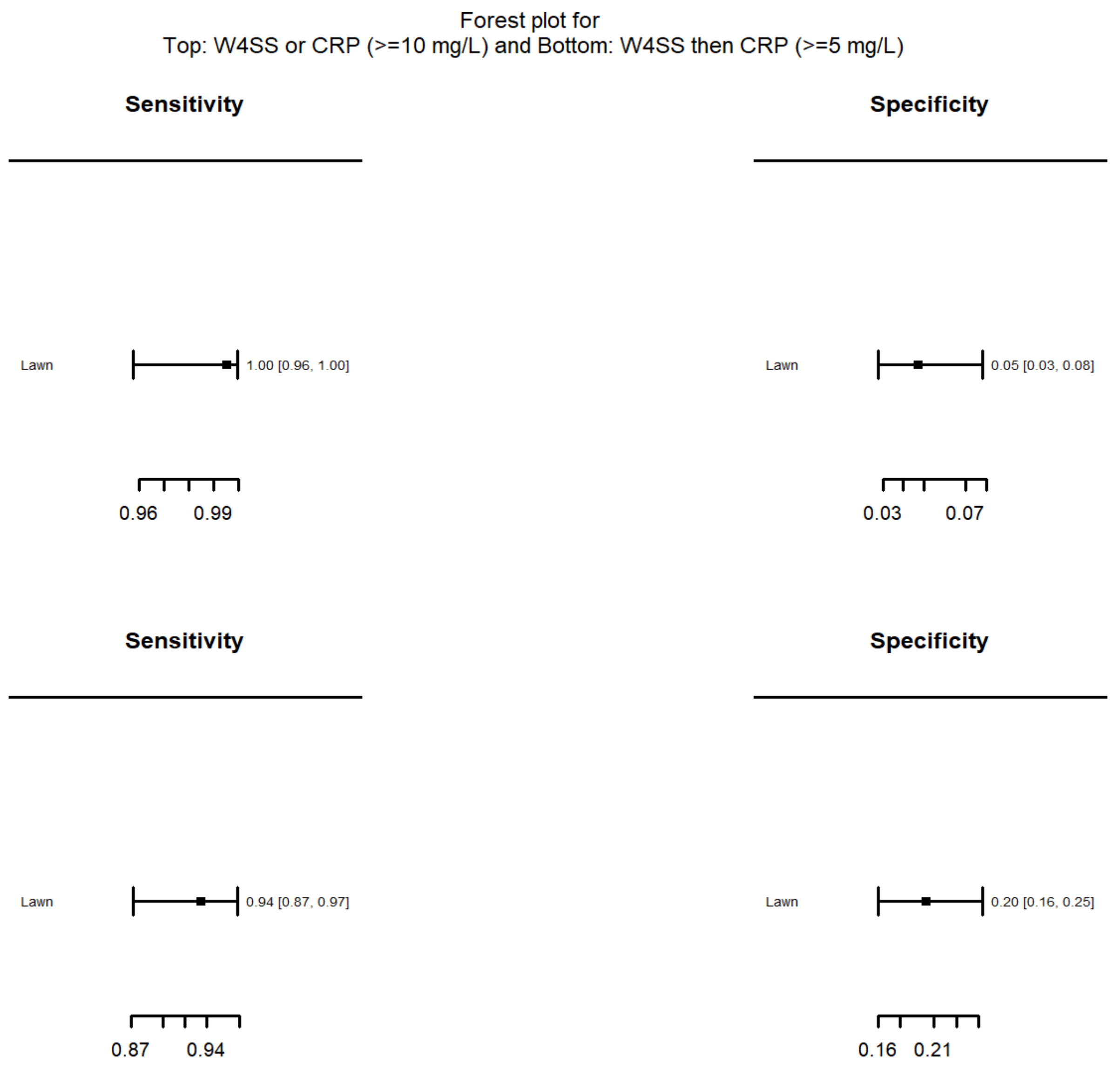
Forest plot for
Top: W4SS or CRP (>=10 mg/L) and Bottom: W4SS then CRP (>=5 mg/L)
Sensitivity
Specificity
Lawn
1.00 [0.96, 1.00]
Lawn
0.05 [0.03, 0.08]
0.96
0.99
0.03
0.07
Sensitivity
Specificity
Lawn
0.94 [0.87, 0.97]
Lawn
0.20 [0.16, 0.25]
0.87
0.94
0.16
0.21

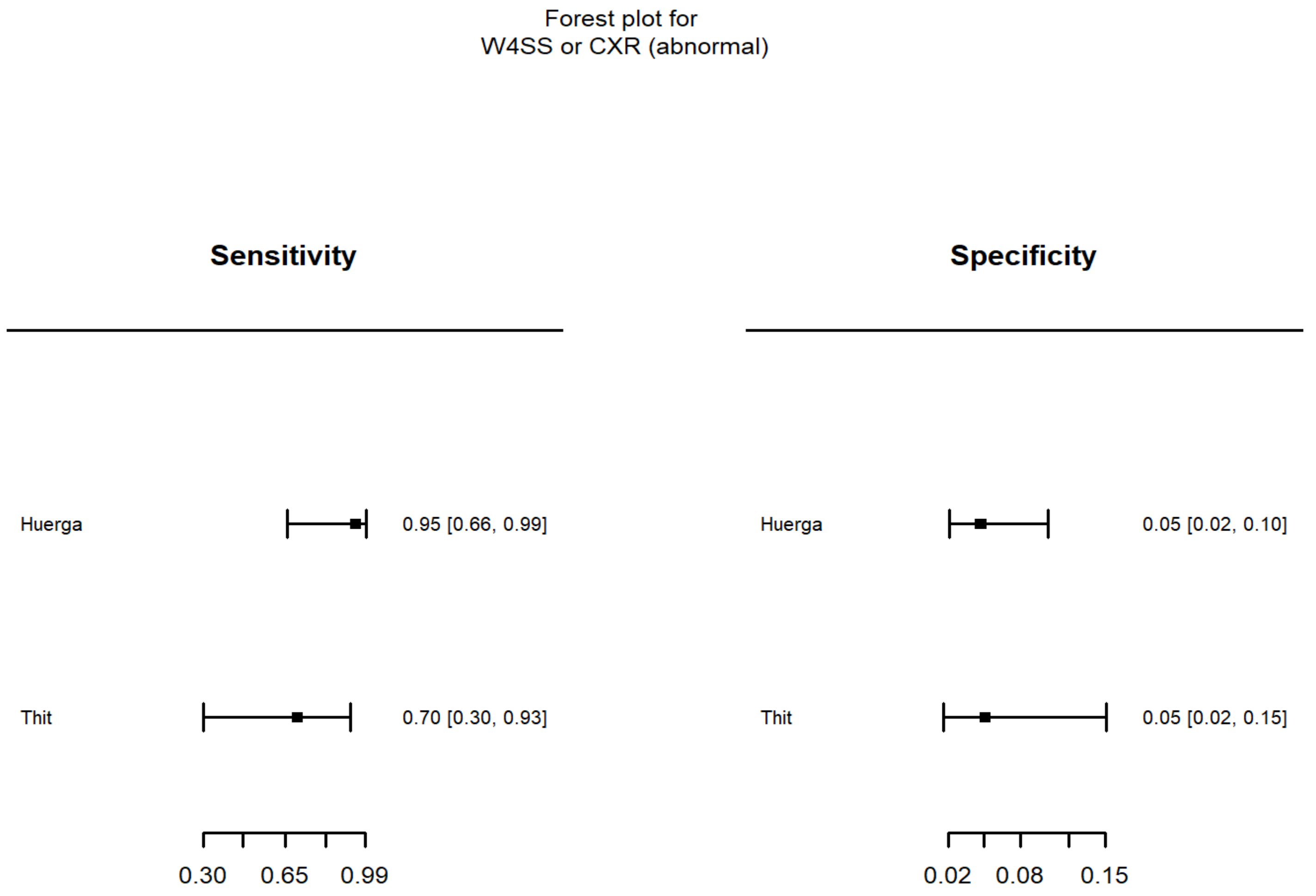
Forest plot for
W4SS or CXR (abnormal)
Sensitivity
Specificity
Huerga
0.95 [0.66, 0.99]
0.05 [0.02, 0.10]
Thit
0.70 [0.30, 0.93]
0.05 [0.02, 0.15]
0.30
0.65
0.99
0.02
0.08
0.15

Figure S2 - Summary receiver operating characteristics curves for each screening test/strategy (for tests/strategies with >=4 studies available)

Figure S2A - Summary receiver operating characteristics curves for each screening test/strategy using culture as a reference standard

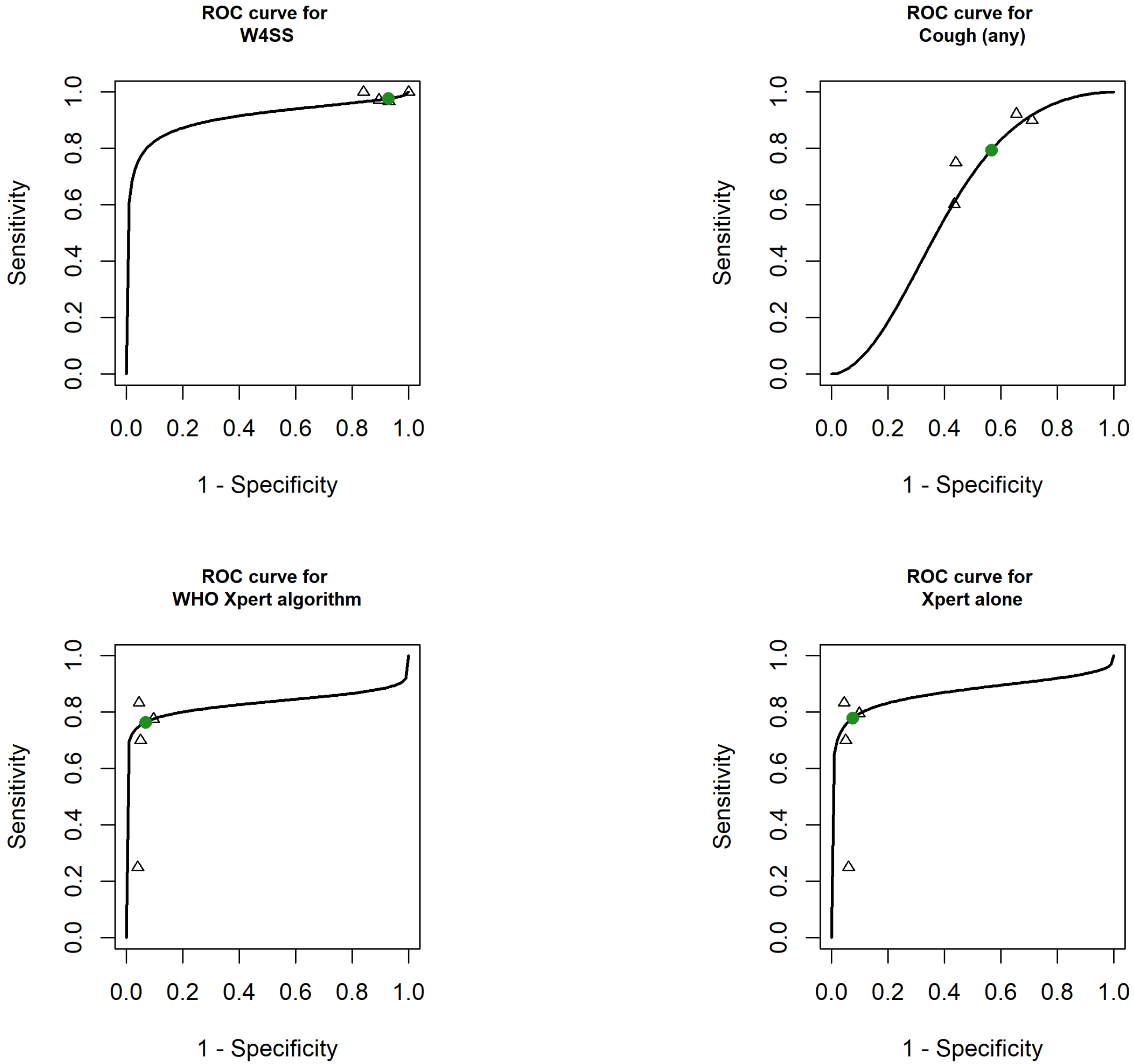

Figure S2B - Summary receiver operating characteristics curves for each screening test/strategy using Xpert as a reference standard

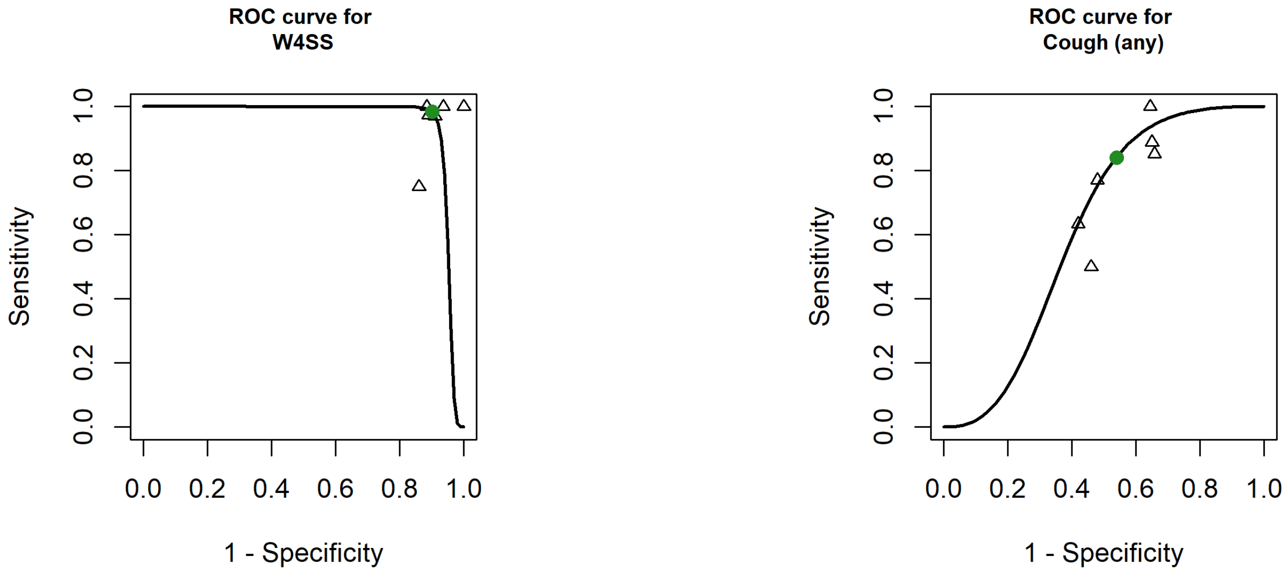

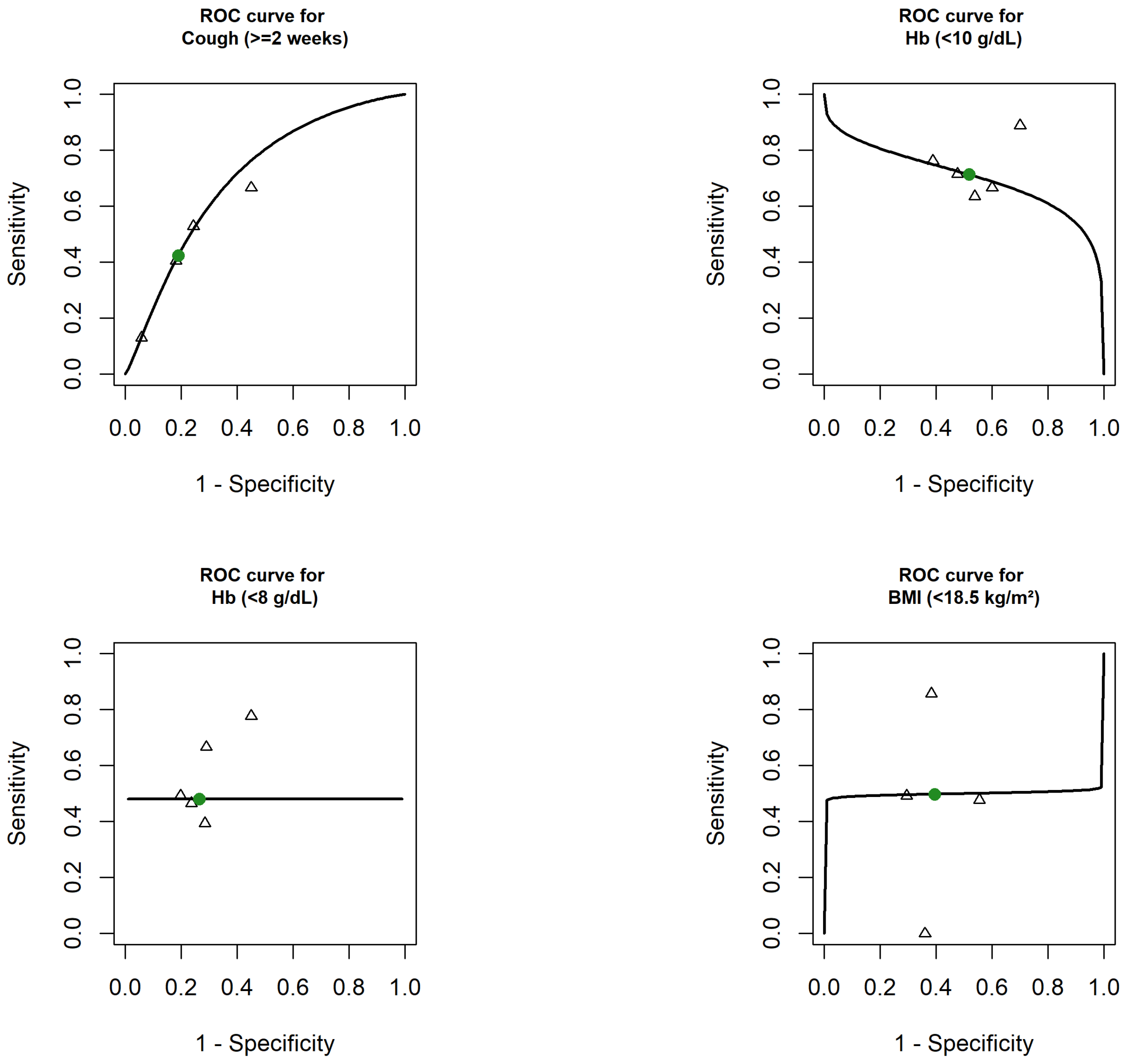
ROC curve for
Cough (>=2 weeks)
ROC curve for
Hb (<10 g/dL)
ROC curve for
Hb (<8 g/dL)
ROC curve for
BMI (<18.5 kg/m²)
Sensitivity
1 - Specificity
0.0
0.2
0.4
0.6
0.8
1.0

Figure S3 - Plot comparing number of tuberculosis cases missed with number of Xpert tests required for different tuberculosis screening tests when screening a population of 1000 persons†

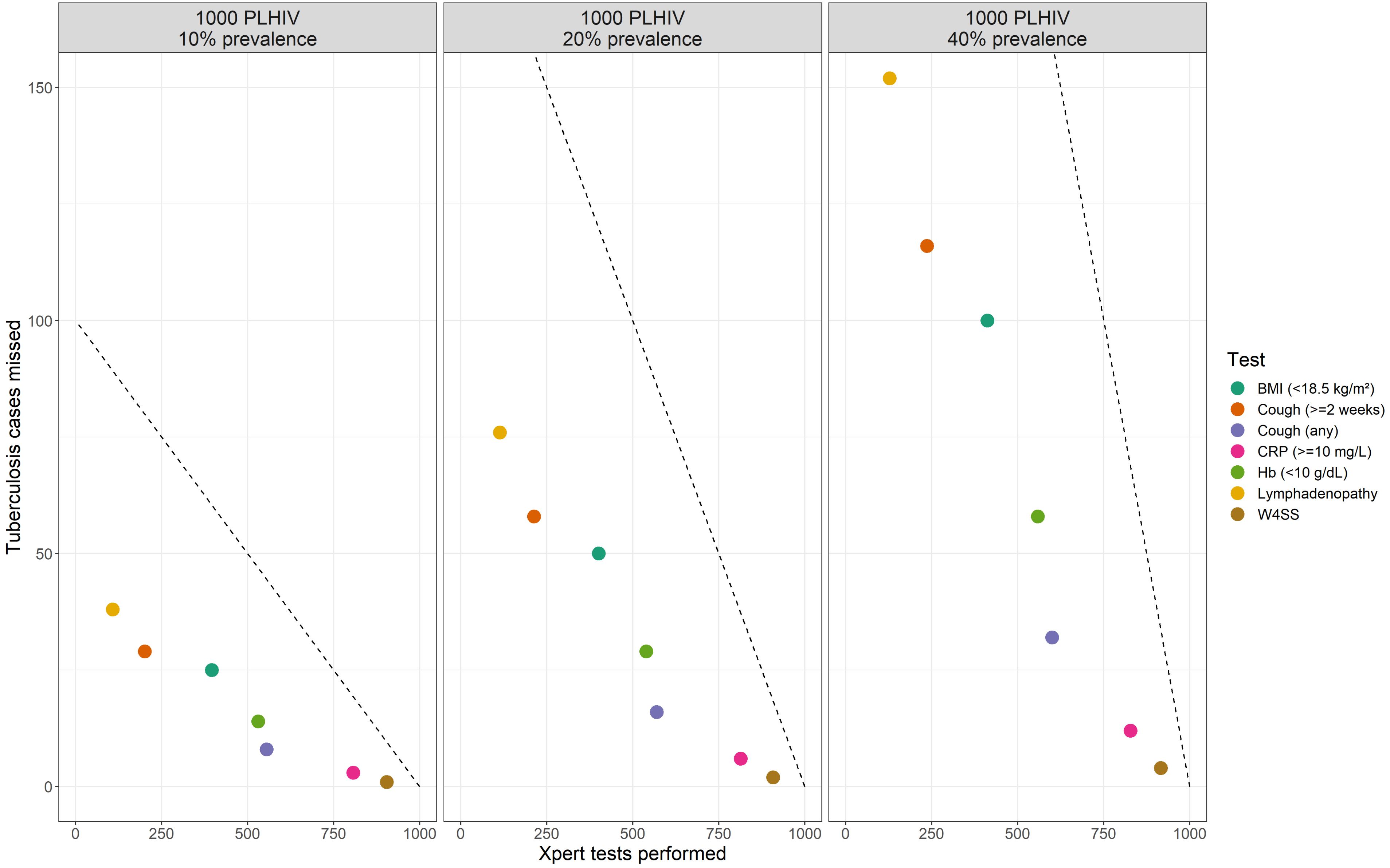

†Using a reference standard of Xpert. The dashed line represents the number of tuberculosis cases diagnosed when applying x Xpert tests at random among 1000 PLHIV. Tests closer to the bottom left corner would offer a better trade-off between tuberculosis cases missed and Xpert tests required
Definition of abbreviations: BMI = body mass index, CRP = C-reactive protein, Hb = haemoglobin, PLHIV = people living with HIV, W4SS = WHO four-symptom screen

References

1. Bjerrum S, Kenu E, Lartey M, et al. Diagnostic accuracy of the rapid urine lipoarabinomannan test for pulmonary tuberculosis among HIV-infected adults in Ghana-findings from the DETECT HIV-TB study. *BMC Infect Dis* 2015; **15**: 407.
2. Gupta-Wright A, Corbett EL, van Oosterhout JJ, et al. Rapid urine-based screening for tuberculosis in HIV-positive patients admitted to hospital in Africa (STAMP): a pragmatic, multicentre, parallel-group, double-blind, randomised controlled trial. *Lancet* 2018; **392**(10144): 292-301.
3. Heidebrecht CL, Podewils LJ, Pym AS, Cohen T, Mthiyane T, Wilson D. Assessing the utility of Xpert((R)) MTB/RIF as a screening tool for patients admitted to medical wards in South Africa. *Sci Rep* 2016; **6**: 19391.
4. Huerga H, Mathabire Rucker SC, Bastard M, et al. Urine Lipoarabinomannan Testing for All HIV Patients Hospitalized in Medical Wards Identifies a Large Proportion of Patients With Tuberculosis at Risk of Death. *Open Forum Infect Dis* 2021; **8**(2): ofaa639.
5. Lawn SD, Kerkhoff AD, Burton R, et al. Rapid microbiological screening for tuberculosis in HIV-positive patients on the first day of acute hospital admission by systematic testing of urine samples using Xpert MTB/RIF: a prospective cohort in South Africa. *BMC Med* 2015; **13**: 192.
6. Thit SS, Aung NM, Htet ZW, et al. The clinical utility of the urine-based lateral flow lipoarabinomannan assay in HIV-infected adults in Myanmar: an observational study. *BMC Med* 2017; **15**(1): 145.

PRISMA-IPD checklist

PRISMA-IPD Checklist of items to include when reporting a systematic review and meta-analysis of individual participant data (IPD)

PRISMA-IPD Section/topic	Item No	Checklist item	Reported on page
**Title**			
Title	1	Identify the report as a systematic review and meta-analysis of individual participant data.	1
**Abstract**			
Structured summary	2	Provide a structured summary including as applicable:	3
		**Background**: state research question and main objectives, with information on participants, interventions, comparators and outcomes.	
		**Methods**: report eligibility criteria; data sources including dates of last bibliographic search or elicitation, noting that IPD were sought; methods of assessing risk of bias.	
		**Results**: provide number and type of studies and participants identified and number (%) obtained; summary effect estimates for main outcomes (benefits and harms) with confidence intervals and measures of statistical heterogeneity. Describe the direction and size of summary effects in terms meaningful to those who would put findings into practice.	
		**Discussion:** state main strengths and limitations of the evidence, general interpretation of the results and any important implications.	
		**Other:** report primary funding source, registration number and registry name for the systematic review and IPD meta-analysis.	
**Introduction**			
Rationale	3	Describe the rationale for the review in the context of what is already known.	5
Objectives	4	Provide an explicit statement of the questions being addressed with reference, as applicable, to participants, interventions, comparisons, outcomes and study design (PICOS). Include any hypotheses that relate to particular types of participant-level subgroups.	5
**Methods**			
Protocol and registration	5	Indicate if a protocol exists and where it can be accessed. If available, provide registration information including registration number and registry name. Provide publication details, if applicable.	5
Eligibility criteria	6	Specify inclusion and exclusion criteria including those relating to participants, interventions, comparisons, outcomes, study design and characteristics (e.g. years when conducted, required minimum follow-up). Note whether these were applied at the study or individual level i.e. whether eligible participants were included (and ineligible participants excluded) from a study that included a wider population than specified by the review inclusion criteria. The rationale for criteria should be stated.	6
Identifying studies - information sources	7	Describe all methods of identifying published and unpublished studies including, as applicable: which bibliographic databases were searched with dates of coverage; details of any hand searching including of conference proceedings; use of study registers and agency or company databases; contact with the original research team and experts in the field; open adverts and surveys. Give the date of last search or elicitation.	6
Identifying studies - search	8	Present the full electronic search strategy for at least one database, including any limits used, such that it could be repeated.	5, 6
Study selection processes	9	State the process for determining which studies were eligible for inclusion.	6

Data collection processes	10	Describe how IPD were requested, collected and managed, including any processes for querying and confirming data with investigators. If IPD were not sought from any eligible study, the reason for this should be stated (for each such study).<br><br>If applicable, describe how any studies for which IPD were not available were dealt with. This should include whether, how and what aggregate data were sought or extracted from study reports and publications (such as extracting data independently in duplicate) and any processes for obtaining and confirming these data with investigators.	6, 7
Data items	11	Describe how the information and variables to be collected were chosen. List and define all study level and participant level data that were sought, including baseline and follow-up information. If applicable, describe methods of standardising or translating variables within the IPD datasets to ensure common scales or measurements across studies.	6, 7
IPD integrity	A1	Describe what aspects of IPD were subject to data checking (such as sequence generation, data consistency and completeness, baseline imbalance) and how this was done.	6, 7
Risk of bias assessment in individual studies.	12	Describe methods used to assess risk of bias in the individual studies and whether this was applied separately for each outcome. If applicable, describe how findings of IPD checking were used to inform the assessment. Report if and how risk of bias assessment was used in any data synthesis.	6, 7
Specification of outcomes and effect measures	13	State all treatment comparisons of interests. State all outcomes addressed and define them in detail. State whether they were pre-specified for the review and, if applicable, whether they were primary/main or secondary/additional outcomes. Give the principal measures of effect (such as risk ratio, hazard ratio, difference in means) used for each outcome.	6, 7
Synthesis methods	14	Describe the meta-analysis methods used to synthesise IPD. Specify any statistical methods and models used. Issues should include (but are not restricted to):<br>• Use of a one-stage or two-stage approach.<br>• How effect estimates were generated separately within each study and combined across studies (where applicable).<br>• Specification of one-stage models (where applicable) including how clustering of patients within studies was accounted for.<br>• Use of fixed or random effects models and any other model assumptions, such as proportional hazards.<br>• How (summary) survival curves were generated (where applicable).<br>• Methods for quantifying statistical heterogeneity (such as I^2 and τ^2).<br>• How studies providing IPD and not providing IPD were analysed together (where applicable).<br>• How missing data within the IPD were dealt with (where applicable).	7
Exploration of variation in effects	A2	If applicable, describe any methods used to explore variation in effects by study or participant level characteristics (such as estimation of interactions between effect and covariates). State all participant-level characteristics that were analysed as potential effect modifiers, and whether these were pre-specified.	7
Risk of bias across studies	15	Specify any assessment of risk of bias relating to the accumulated body of evidence, including any pertaining to not obtaining IPD for particular studies, outcomes or other variables.	7
Additional analyses	16	Describe methods of any additional analyses, including sensitivity analyses. State which of these were pre-specified.	7
**Results**			
Study selection and IPD obtained	17	Give numbers of studies screened, assessed for eligibility, and included in the systematic review with reasons for exclusions at each stage. Indicate the number of studies and participants for which IPD were sought and for which IPD were obtained. For those studies where IPD were not available, give the numbers of studies and participants for which aggregate data were available. Report reasons for non-availability of IPD. Include a flow diagram.	8
Study characteristics	18	For each study, present information on key study and participant characteristics (such as description of interventions, numbers of participants, demographic data, unavailability of outcomes, funding source, and if applicable duration of follow-up). Provide (main) citations for each study. Where applicable, also report similar study characteristics for any studies not providing IPD.	8

IPD integrity	A3	Report any important issues identified in checking IPD or state that there were none.	8
Risk of bias within studies	19	Present data on risk of bias assessments. If applicable, describe whether data checking led to the up-weighting or down-weighting of these assessments. Consider how any potential bias impacts on the robustness of meta-analysis conclusions.	8
Results of individual studies	20	For each comparison and for each main outcome (benefit or harm), for each individual study report the number of eligible participants for which data were obtained and show simple summary data for each intervention group (including, where applicable, the number of events), effect estimates and confidence intervals. These may be tabulated or included on a forest plot.	8, 9
Results of syntheses	21	Present summary effects for each meta-analysis undertaken, including confidence intervals and measures of statistical heterogeneity. State whether the analysis was pre-specified, and report the numbers of studies and participants and, where applicable, the number of events on which it is based.	8, 9
		When exploring variation in effects due to patient or study characteristics, present summary interaction estimates for each characteristic examined, including confidence intervals and measures of statistical heterogeneity. State whether the analysis was pre-specified. State whether any interaction is consistent across trials.	
		Provide a description of the direction and size of effect in terms meaningful to those who would put findings into practice.	
Risk of bias across studies	22	Present results of any assessment of risk of bias relating to the accumulated body of evidence, including any pertaining to the availability and representativeness of available studies, outcomes or other variables.	8
Additional analyses	23	Give results of any additional analyses (e.g. sensitivity analyses). If applicable, this should also include any analyses that incorporate aggregate data for studies that do not have IPD. If applicable, summarise the main meta-analysis results following the inclusion or exclusion of studies for which IPD were not available.	8, 9
**Discussion**			
Summary of evidence	24	Summarise the main findings, including the strength of evidence for each main outcome.	9
Strengths and limitations	25	Discuss any important strengths and limitations of the evidence including the benefits of access to IPD and any limitations arising from IPD that were not available.	10, 11
Conclusions	26	Provide a general interpretation of the findings in the context of other evidence.	11
Implications	A4	Consider relevance to key groups (such as policy makers, service providers and service users). Consider implications for future research.	11
**Funding**			
Funding	27	Describe sources of funding and other support (such as supply of IPD), and the role in the systematic review of those providing such support.	12

A1 – A3 denote new items that are additional to standard PRISMA items. A4 has been created as a result of re-arranging content of the standard PRISMA statement to suit the way that systematic review IPD meta-analyses are reported.

© Reproduced with permission of the PRISMA IPD Group, which encourages sharing and reuse for non-commercial purpose
